# A review of *Calypogeia* (Marchantiophyta) in the eastern Sino-Himalaya and Meta-Himalaya based mostly on types

**DOI:** 10.3897/phytokeys.153.52920

**Published:** 2020-07-16

**Authors:** Vadim A. Bakalin, Ksenia G. Klimova, Van Sinh Nguyen

**Affiliations:** 1 Botanical Garden-Institute, Vladivostok, Russia Botanical Garden-Institute Vladivostok Russia; 2 Institute of Ecology and Biological Resources, Graduate University of Science and Technology, Vietnam Academy of Science and Technology, Ha Noi, Vietnam University of Science and Technology Ha Noi Vietnam

**Keywords:** Calypogeiaceae, East Asia, Hepaticae, Indochina, taxonomy, typification

## Abstract

The eastern part of the southern macroslope of the Himalayan Range, Hengduan Mountains and the complex of smaller ranges from Hengduan southward to northern Indochina is one of the taxonomic hotspots of *Calypogeia* in Asia and the world. Two main circumstances hamper the understanding of taxonomic diversity of the genus in this area: the absence of recent and detailed descriptions and identification keys and the necessity of studying fresh material with surviving oil bodies in leaf cells. This study resulted in 1) eleven species confirmed for this vast land, 2) seven more taxa recorded but likely based on identification mistakes and 3) fourteen more taxa that are not yet recorded but may be expected in the area. All these taxa are discussed, and most of them are illustrated and described based on the types; an identification key is provided. The occurrence of North Holarctic taxa is hardly probable in the Sino-Himalaya, whereas new records of taxa known from the southern half of the Japanese Archipelago, Taiwan and southeastern mainland China are possible.

## Introduction

*Calypogeia* in East Asia has attracted only slight special attention from hepaticologists. Despite some recent advances in the systematic analysis of the genus based on the study of East Asian material (Buczkowska et al. 2018; Bakalin et al. 2019a), *Calypogeia* should still be regarded as a group that is very difficult to identify, the taxonomy of which is hardly understood. There are two basic reasons for this outstanding difficulty: 1) the absence of reliable keys for identification and 2) the necessity of studying the living material to observe oil body characteristics. Meanwhile, if even fresh material is available and oil body characteristics are studied, the problem remains of where to place the material if almost no oil body characteristics are indicated in the descriptions (both original or based on other materials) and the keys. Therefore, the aforementioned reasons are distinctly related. The attempts to identify East Asian *Calypogeia* using European keys are questionable: 1) the records of some taxa occurring in the Sino-Himalaya should be strongly doubted, and 2) the synonymization of some Himalayan taxa with European analogs was hasty. Understandably, to some extent, this situation was provoked by the brevity of original descriptions in the literature of the second half of the 19th century and the first quarter of the 20th century.

The basic and inevitable tasks for progress in the knowledge of *Calypogeia* in the southern part of the East Asian floristic region should be the compilation of morphological descriptions as detailed as possible based on the study of types and the compilation of an identification key to the *Calypogeia* taxa recorded or expected in this area. The two tasks are the main goals of the present study.

## Material and methods

The vast majority of *Calypogeia* based on material originating from the Sino-Himalaya, in the broad sense, were described by W. Mitten and F. Stephani. Fortunately for our purposes, Stephani also largely duplicated Mitten’s collection, and many type materials (including syntypes, isotypes and isolectotypes) are now housed in G (acronyms follow Thiers 2020). The material from G therefore has irrefutable value for our work, and most of the studied material is from there, although some additional specimens were studied in JE, NICH, STR, TNS and VBGI. In total, 43 types were studied, and many of them were photographed and illustrated. The taxonomic part of the work has the following subchapters, determined by practical reasons:

1) Taxa undoubtedly occurring in the study area (e.g., if the type specimen is from there).

2) Doubtful records of taxa that have a very low probability of being observed there.

3) Taxa that are not recorded in the study area but may be expected. We treat the latter definition very broadly, as involving some taxa from as far as India and Japan that look reasonable taking into account, e.g., the undoubted occurrence of *Calypogeia granulata* – a formerly Japanese endemic taxon – in Guizhou Province, China, confirmed by molecular genetic research (Buczkowska et al. 2018).

4) A dichotomous key to the taxa observed (also including doubtful records) and expected in the study area.

Each taxon in the taxonomic section is annotated as usual, with data on studied type specimens, morphological description based on the type and other comments on morphology or ecology. When providing the distribution of taxa, we do not limit the data to the study area only, but largely also include data from other, nearby regions or areas that have distinct relations in mountain flora with the Sino-Himalaya, e.g., the mountain flora of Taiwan.

The valuable problem of the present work is the inability to evaluate the morphological variation parameters clearly for many species; since there are only a few specimens known (e.g. *Calypogeia marginella* is known from the type gathering only). In these cases, we accepted ‘narrow species concept’, to avoid the loss of information resulting from hasty synonymization and, therefore, to keep by now as much taxa accepted as possible. In addition, we followed general estimations on the morphological variability of taxa in *Calypogeia* obtained in our previous works in this group (Buczkowska et al. 2018; Bakalin et al. 2019a).

## Study area

It is quite difficult to describe the ‘Sino-Himalaya’ using definite terminology. In very general terms, it is a large territory including the Himalaya Range with some spurs as well as mountain ranges in Southwest China, where it generally includes the Hengduan Shan – a very unclear term for the large mountain massif stretching from the Tibetan Plateau to the southeast until intersection with the mountainous northern end in Indochina. Despite the unclear definition, “the biogeographic unit informally known as the ‘Sino-Himalayan region’” (Váňa and Long 2009: 487) was widely used in the literature starting from the beginning of the 20th century. Váňa and Long identified the Sino-Himalaya in its common sense, including “Pakistan Himalaya, Indian Himalaya (Jammu & Kashmir, Himachal Pradesh, Uttaranchal, Sikkim, Darjeeling District of West Bengal, Assam, Meghalaya, Manipur and Arunachal Pradesh), Nepal, Bhutan and western China (Yunnan, Sichuan and Xizang (Tibet))” (Váňa and Long 2009: 487). A similar view was maintained by many botanists, including bryologists (e.g., Dalton et al. 2013). The accepted above treatment of the Sino-Himalaya does not mean that this is a monomorphous and floristically indivisible unit. The strong and noticeable differentiation along the longitudinal gradient was evident even at the beginning of the 20th century, when several new expeditions explored this land more carefully than before. Moreover, even at this time, it was evident that some distant regions in the Sino-Himalaya have more common species than some nearer ones (Ward 1925). Ward (1925) also noted the possible wide spread of Sino-Himalayan taxa by rivers going in very diverse directions, from the Brahmaputra in the west (draining to the Bay of Bengal) to the Yangtze River in the east (making a strong curve in southern Hengduan and then draining to the East China Sea), with many large rivers between, such as the Mekong River (draining to southernmost Indochina). Ward (1921) identified the watershed between the Mekong and Salween Rivers as an important phytogeographic boundary.

The eastern Sino-Himalaya is identified here as the land included in the Sino-Himalaya eastward of eastern Nepal. The Meta-Himalaya is identified as an area surrounding the southeastern part of the Sino-Himalaya, although not belonging to the Sino-Himalaya in its common sense. It includes eastern Sichuan, western Guizhou, eastern Yunnan and the mountains of northern Indochina. This is an area where Sino-Himalayan species deeply penetrate, although sometimes represented by transformed races or the speciation derivates of species status (liverwort examples are in Bakalin et al. 2018, 2019a). This broad definition is more natural than may be expected from superficial examination. The deep relationships, e.g., between the floras of western Sichuan and northern Vietnam, were stressed by Takhtajan (1978, 1986). Chen et al. (2018) subdivided East Asia (treated by them as a plant kingdom) into two ‘subkingdoms’, conditionally calling them the *Rhododendron* flora and the *Metasequoia* flora, where the *Rhododendron* flora is somewhat related to the eastern Sino-Himalaya until it mildly contacts the *Metasequoia* flora along a line through the middle of Sichuan and Guizhou Provinces in China. Neither the *Rhododendron* subkingdom nor the *Metasequoia* subkingdom of East Asia extend southward to the Indochina Peninsula (Chen et al. 2018), despite the vegetation at upper elevations in the mountains of northernmost Vietnam not being Paleotropic. Indeed, Averyanov et al. (2003: 74) provided evidence that the characteristic flora at elevations above 1400 m a.s.l. even slightly southward of Phan Xi Pang Mt. “approximates the floras typical for the Sikang-Yunnan floristic province of the Holarctic floristic kingdom”.

The area treated in this work covers four floristic provinces in the sense of Takhtajan (1978, 1986):

1) Sikang-Yunnan floristic province that covers western Sichuan, western Yunnan, northeastern Myanmar, northern Laos and northwestern Vietnam (including the Hoang Lien Range). Takhtajan (1978, 1986) stressed that this large province undoubtedly should be split into several independent provinces in the future when new data are available.

2) North Burma (= Myanmar) floristic province.

3) East Himalayan province, including eastern Nepal (excluding low elevations with tropical vegetation), Darjeeling, Sikkim, Bhutan, the Assam Himalaya and the southern and southeastern flanks of Xizang (Tibet), where the monsoon climate is still pronounced. Takhtajan (1978) also noted the absence of a sharp floristic border in the eastern part, where it gradually transmutes into the central Chinese province.

4) Eastern part of the central Chinese province (western Guizhou, eastern Sichuan and eastern Yunnan).

We do not include Chinese Tibet (Xizang) to the eastern Sino-Himalaya as was done, e.g., by Váňa and Long (2009) and Dalton et al. (2013) because of the strong difference in the vegetation and taxonomic composition compared with other parts of the eastern Sino-Himalaya. Moreover, the Tibetan Plateau is part of other floristic region (Irano-Turanian, cf. Takhtajan 1978) characterized by the dominance of relatively younger taxa of dry Central Asian or even ancient Mediterranean origin.

**Figure 1.**
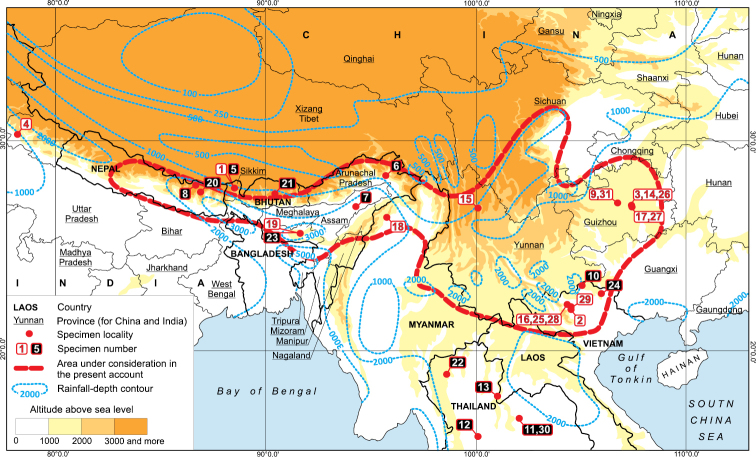
The area considered in the present paper. *Calypogeia aeruginosa* (**1, 2**), *Calypogeia angusta* (**3**), *Calypogeia apiculata* (**4**), *Calypogeia arguta* (**4–14**), *Calypogeia cordistipula* (**15**), *Calypogeia granulata* (**16, 17**), *Calypogeia lunata* (**18–22**), *Calypogeia marginella* (**23**), *Calypogeia tosana* (**24–26**), *Calypogeia sinensis* (**27, 28**), *Calypogeia vietnamica* (**29**), *Calypogeia goebelii* (**30**), *Calypogeia japonica* (**31**). White solid squares – specimens examined, black solid squares – specimens not seen.

To identify the general character of the vegetation in the study area, this is alpine vegetation in forest-free landscapes at high elevations extending down to the vegetation developed above tropical communities, starting from the mountain subtropics. The Sino-Himalaya is dominated by a strong monsoon climate and has a distinct cool season with at least occasional snowfall, even at the southern extremes, such as peaks of the Hoang Lien Range. The area under consideration is depicted in Fig. 1.

## Taxonomic treatment

### Taxa confirmed in the area

#### Calypogeia aeruginosa

Taxon classificationPlantaeJungermannialesCalypogeiaceae

Mitt., J. Proc. Linn. Soc., Bot. 5 (18): 107. 1860 [1861].

9A519AEC-0AE8-51BE-ADC6-39CDEC3EC632

Figure 2A

##### Type.

India. Sikkim: 12000 ped. alt. (4000 m a.s.l.), J.D. Hooker, no. 1319 (isotype: G [G00064244/5286!]).

##### Remarks.

This taxon is broadly Sino-Himalayan-Taiwan-Japanese endemic, distributed within this large area quite disjunctively (although this may reflect the data deficiency). It was described from Sikkim (Mitten 1860), later recorded from Taiwan (Wang et al. 2011) and eastward from southernmost Japan (Inoue 1969; Yamada and Iwatsuki 2006). A questionable record is from Hawaii under the name *Calypogeia waialealeensis* (H.A. Mill. & Kuwah.) H.A. Mill. (Miller 1967) – the name synonymized with *C. aeruginosa* by Inoue (1969). From the geographic point of view, this synonymization should be doubted and the status of the populations from Hawaii should be rechecked, including molecular-genetic methods implementing. We recently found *C. aeruginosa* in northern Vietnam (Buczkowska et al. 2018) which may imply its broader distribution in the Meta-Himalaya. The taxon is very distinctive among congeners due to transversely elliptic underleaves as large as or larger than leaves and may be rather mistaken at the time of collection for *Leucolejeunea* due to size, color, leaf orientation and large underleaves (obscuring the fact that the lejeuneaceous lobule is absent here).

**Figure 2.**
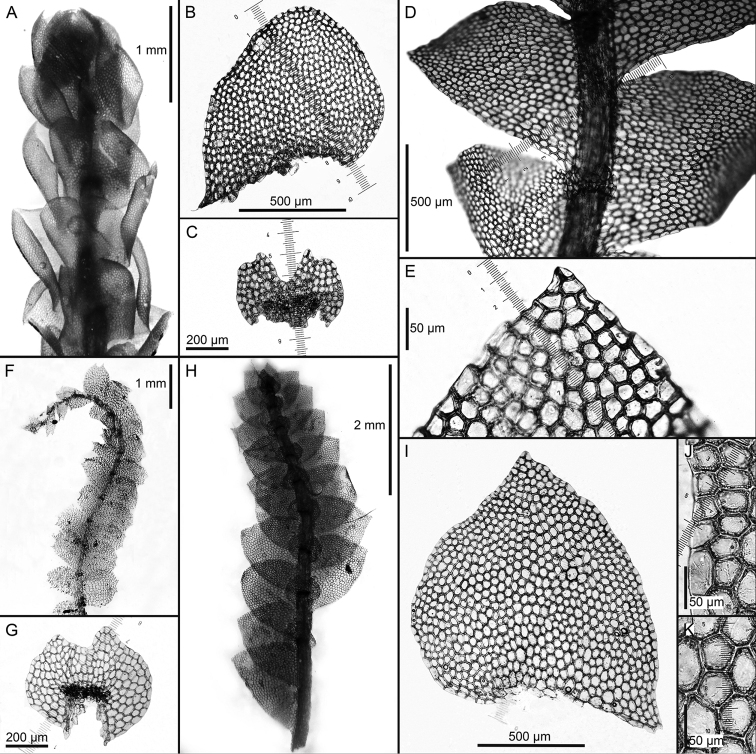
*Calypogeia aeruginosa* Mitt.: **A** plant habit, fragment, ventral view *Calypogeia angusta* Steph.: **B** leaf **C** underleaf *Calypogeia apiculata* (Steph.) Steph.: **D** plant habit, fragment, ventral view **E** leaf apex *Calypogeia arguta* Nees et Mont.: **F** plant habit, fragment, ventral view *Calypogeia cordistipula* (Steph.) Steph.: **G** underleaf **H** plant habit, fragment, ventral view **I** leaf **J** leaf margin cells **K** leaf middle cells. Scale bars: 1 mm (**A, F**); 500 µm (**B, D, G**); 200 µm (**C, G**); 50 µm (**E, J, K**); 2 (**N**); 2 mm (**H**). **A** from Isotype G00064244/5286; **B, C** from Lectotype G00067716; **D, E** Holotype G00061103; **F** Holotype STR (n. 163); **G–K** from Lectotype G00061105/10811.

#### Calypogeia angusta

Taxon classificationPlantaeJungermannialesCalypogeiaceae

Steph., Bull. Herb. Boissier (sér. 2) 8 (9): 663 (395). 1908.

7B2E82B5-CB06-5434-B711-C736199CEC78

**Figure 3.**
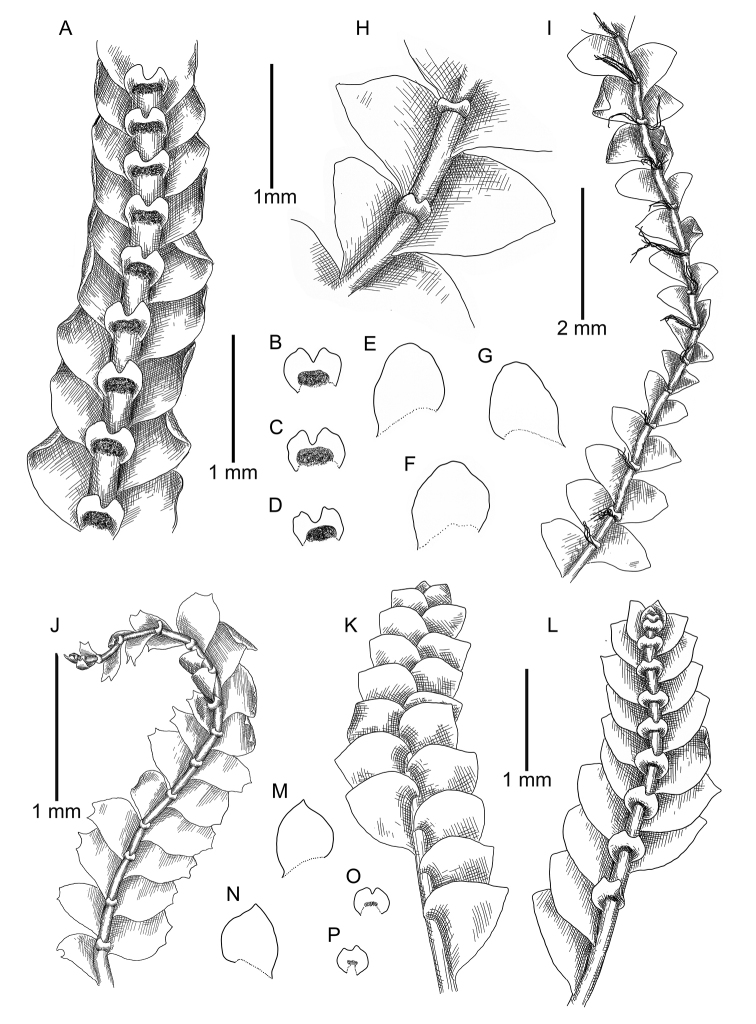
*Calypogeia angusta* Steph.: **A** plant habit, fragment, ventral view **B, C, D** underleaves **E, F, G** leaves *Calypogeia apiculata* (Steph.) Steph.: **H** plant habit, fragment, ventral view **I** plant habit, fragment, ventral view *Calypogeia arguta* Nees et Mont.: **J** plant habit, fragment, ventral view *Calypogeia cordistipula* (Steph.) Steph.: **K** plant habit, fragment, dorsal view **L** plant habit, fragment, ventral view **M, N** leaves **O, P** underleaves. **A–G** from Lectotype G00067716; **H, I** lectotype G00061103; **J** holotype STR (n. 163); **K–P** from Lectotype G00061105/10811.

Figures 2B, C , 3A–G

##### Type.

Japan. Ozoresan: 11 October 1902, U. Faurie, 1181 (Lectotype (designated here): G [G00067716!]).

##### Remarks.

The species was described in Japan (Stephani 1908) and was recently recorded in Chinese Guizhou (Bakalin et al. 2015). We may suggest that some reports of *Calypogeia muelleriana* from China may likely belong to this taxon due to rounded leaf lobes; if the underleaves are not considered, they are much more deeply divided and commonly bisbifid. The description based on the lectotype is as follows: plants more or less rigid, barely translucent, slightly glistening, yellowish brownish in the herbarium, well-developed plants 900–1700 µm wide; stem 200–300 µm wide, sparsely ventrally branched; rhizoids in obliquely spreading, brownish fascicles; leaves contiguous to subimbricate (overlapping 2/5 of adjacent leaf), obliquely inserted and oriented, convex, with apices turned to the ventral side, obliquely ovate, apex rounded, entire to somewhat crispate, 500–750 × 500–800 µm; underleaves 1.1–1.4 as wide as stem, decurrent for ¼ of stem width or less, bisbifid or with one additional lateral tooth on each side, sinus V-shaped, undivided portion 1–2 cell high; midleaf cells 25–55 × 25–37 µm, thin walled with small to moderate size, concave trigones, cuticle smooth.

#### Calypogeia apiculata

Taxon classificationPlantaeJungermannialesCalypogeiaceae

(Steph.) Steph., Bull. Herb. Boissier (sér. 2) 8 (9): 668 (400). 1908.

5CF7DD11-D8AE-58FA-A4BE-AC3F80DE4AF5

Figures 2D, E ; 3H, I

= Calypogeia gollanii Steph. ex Bonner Index Hepaticarum, 1963. nom. inval. (Art. 38.1(a); no description). Authentic material (invalid names have no types): India. NW Himalaya: Mussoorie W. Gollan 01 Nov 1900 (original material, probably scheduled as the type): G [G00067720/23987!^*^] Syn. nov.

##### Basionym.

*Kantius apiculatus* Steph., Hedwigia 34 (2): 51, 1895.

##### Type.

Java. Prof. Stahl (Lectotype (designated here): G [G00061103!]).

##### Remarks.

The species was originally described from Java (Stephani 1895) and recorded from Borneo (Chuah-Petiot 2011) and Sri Lanka (Long and Rubasinghe 2014). *Calypogeia gollanii* in India is the synonym of *C. apiculata*. Although *Calypogeia gollanii* is regarded as the synonym of *C. azurea* in the https://bryophyteportal.org/, the plants correspond in all ways to *C. apiculata* Steph., although the leaf cuticle is very loosely (although obviously) papillose. *Calypogeia azurea* does not occur in East Asia (Buczkowska et al. 2018) and has much wider underleaves. *Calypogeia apiculata* may also be mistaken for *C. sphagnicola* (due to small underleaves) – generally *Sphagnum* swamp species that could hardly be expected in the Sino-Himalaya. In addition, *Calypogeia sphagnicola* has highly distanced and smaller leaves and smooth leaf cuticles. Presumably, the reports of *C. sphagnicola* in China may actually represent *C. apiculata*.

The description based on the lectotype of *C. apiculata* is as follows: plants 1.0–2.2 mm wide, 3–5 cm long, pale yellowish brownish in the herbarium; stem ~180 µm wide; rhizoids sparse to numerous in obliquely spreading fascicles, leaves distant to contiguous, nearly planar to slightly convex, rarely incurved to dorsal side (probably due to long drying and repeated soaking), 600–1100 × 450–800 µm, obliquely ovate, apiculate, very rarely shortly bidentate, decurrent in ventral base for 0.5–1.0 of stem width; underleaves as wide as stem or slightly wider, bilobed, undivided portion (1–)2 cells high, lateral teeth absent, decurrent for 1/3 of stem width or less; cuticle in leaves and underleaves very finely verruculose; cells in the midleaf 37–58 × 25–35 µm, thin-walled, trigones very small and concave.

#### Calypogeia arguta

Taxon classificationPlantaeJungermannialesCalypogeiaceae

Nees et Mont., Naturgesch. Eur. Leberm. 3: 24. 1838.

05A9A4D0-882A-5231-88C5-9855E5B508F7

Figures 2F , 3J

= Calypogeia pusilla Steph. Species Hepaticarum 6: 450. 1924. Type: India. India Orientalis: Madura A. Vella 1910 (Lectotype (designated here): G [G00067728/10974!].

##### Type.

Montagne (holotype: STR [(n. 163)!]).

##### Remarks.

The species is described in “südlichen Frankreich, auf der Erde” (Nees 1838: 24), has generally suboceanic-Mediterranean (Damsholt 2002: 460) distribution, is widely distributed in Mediterranean areas in southern Europe (hardly spreading northward to Nordic countries) and North Africa, widely penetrates Asia along areas of the former Tethys Ocean surroundings and extends eastward to New Guinea; within North America, it is substituted by *Calypogeia sullivantii* Austin, a morphologically very similar taxon. In the genetic sense, this polymorphous taxon probably includes several cryptic or semicryptic species. *Calypogeia pusilla*, described from Indian Madura, represents in morphological respects the only depauperate form of typical *C. arguta*.

Within East Asia, *Calypogeia arguta* is recorded from Assam, Sikkim, (Robinson 1964; Bapna and Kachroo 2000), eastern Nepal (Noguchi et al. 1966), several localities in China, namely, Guangxi (Zhu and So 2003), Hong Kong (Zhang and Lin 1997), Jiangxi (Fang et al. 1998), Jiangsu, Guangdong, Hainan, Taiwan (Piippo 1990), Liaoning, Shaanxi, Shandong, Hubei, Yunnan, Henan, Anhui, Zhejiang, Hunan, Fujian, Guangxi, Macau (http://www.catalogueoflife.org/annual-checklist/2019/), and Guizhou Provinces (Bakalin et al. 2015). At the northern edge of East Asia the species is recorded from Kuril Islands (Bakalin et al. 2009), Japan and Korean Peninsula (http://www.catalogueoflife.org/annual-checklist/2019/). In Southeast Asia it is known from Vietnam (Shu et al. 2017), Thailand, Andaman Islands, Nicobar Is, Malaya, Borneo, Sulawesi, Java (http://www.catalogueoflife.org/annual-checklist/2019/).

#### Calypogeia cordistipula

Taxon classificationPlantaeJungermannialesCalypogeiaceae

(Steph.) Steph. Species Hepaticarum 3: 400. 1908.

17393B03-C974-59A8-B545-D4662CB96AD0

Figures 2G–K , 3K–P

= Cincinnulus cordistipulus Steph. Mémoires de la Société des Sciences Naturelles et Mathématiques de Cherbourg 29: 210. 1894.

##### Type.

China. Yunnan: Hokin Delavay, no 1623 (Lectotype (designated here): G [G00061105/10811!]).

##### Remarks.

*Calypogeia cordistipula* (Steph.) Steph was reported by Piippo (1990) for Chinese Yunnan (based on the type) but later synonymized with *C. neesiana* (Piippo et al. 1997). However, the two species are quite different. The differences from *C. neesiana* include acute leaf apex, slightly larger cells, acute sinus in underleaves, absence of distinctly elongated cells along leaf margin, no pale coloration (in the present specimen, we suspect blue oil bodies because plants become somewhat blackish-grayish as is common for blue-oil-bodied taxa). We suggest that *C. neesiana* does not occur in the Sino-Himalaya and that all records of that species may actually belong to *C. cordistipula*.

The description based on the lectotype is as follows: plants greenish brownish to grayish brown, 1.5–2.1 mm wide, translucent, slightly glistening; stem ~2500 µm wide, branching not seen; rhizoids common, in brownish fascicles erect to upward obliquely spreading; leaves obliquely inserted, subhorizontally oriented, overlapping 1/2 of the next leaf in the base, loosely concave to almost planar, with apex slightly turned to dorsal side, not or for 1/3 of stem width decurrent, 800–1200 × 800–1200 µm, obliquely widely ovate-triangular, apex acute, never divided; underleaves appressed to the stem to obliquely spreading, 1.8–2.5 as wide as stem, decurrent for 1/2–2/3 of stem width, divided by U- to V-shaped sinus into two lobes without additional teeth, lobes obtuse, undivided portion 3–5 cells high; midleaf cells 40–50 × 40–68 µm, thin-walled, trigones small to very small, concave, cuticle virtually smooth.

#### Calypogeia granulata

Taxon classificationPlantaeJungermannialesCalypogeiaceae

Inoue, J. Jap. Bot. 43 (10/11): 468. 1968.

4D3BB26D-31A0-5EDD-A4ED-7711115424DC

Figures 4A–K , 5A–E

##### Type.

Japan. Saitama Prefecture: Kuroyama, 500 m a.s.l., 24 June 1968 H. Inoue 18004 (holotype: TNS [174361!]; isotype: G [G00114896!]).

*Calypogeia granulata* was previously treated as a Japanese endemic taxon. Later, however, it was recorded (also confirmed by DNA testing) for northern Vietnam and Guizhou Province in China (Buczkowska et al. 2018; Bakalin et al. 2018). Moreover, strong infraspecific genetic variation was observed within the taxon. It is worth mentioning that some Japanese populations are farther from the type that was also sequenced than the genetic distance between the type and the accessions from Guizhou and Vietnam (cf. Buczkowska et al. 2018). Two specimens named *C. granulata* from Japan (Buczkowska et al. 2018) are so well distanced from the bulk of other so-named specimens that they may be regarded as discrete subspecies (if not separate species!). The variation in oil body color was additionally observed in the species. The taxon was described as having blue-grayish oil bodies, but oil bodies are totally gray to grayish in Guizhou material. Whether these colors represent the stage preceding morphological deterioration or a real morphological peculiarity of geographically distanced populations is currently unknown.

**Figure 4.**
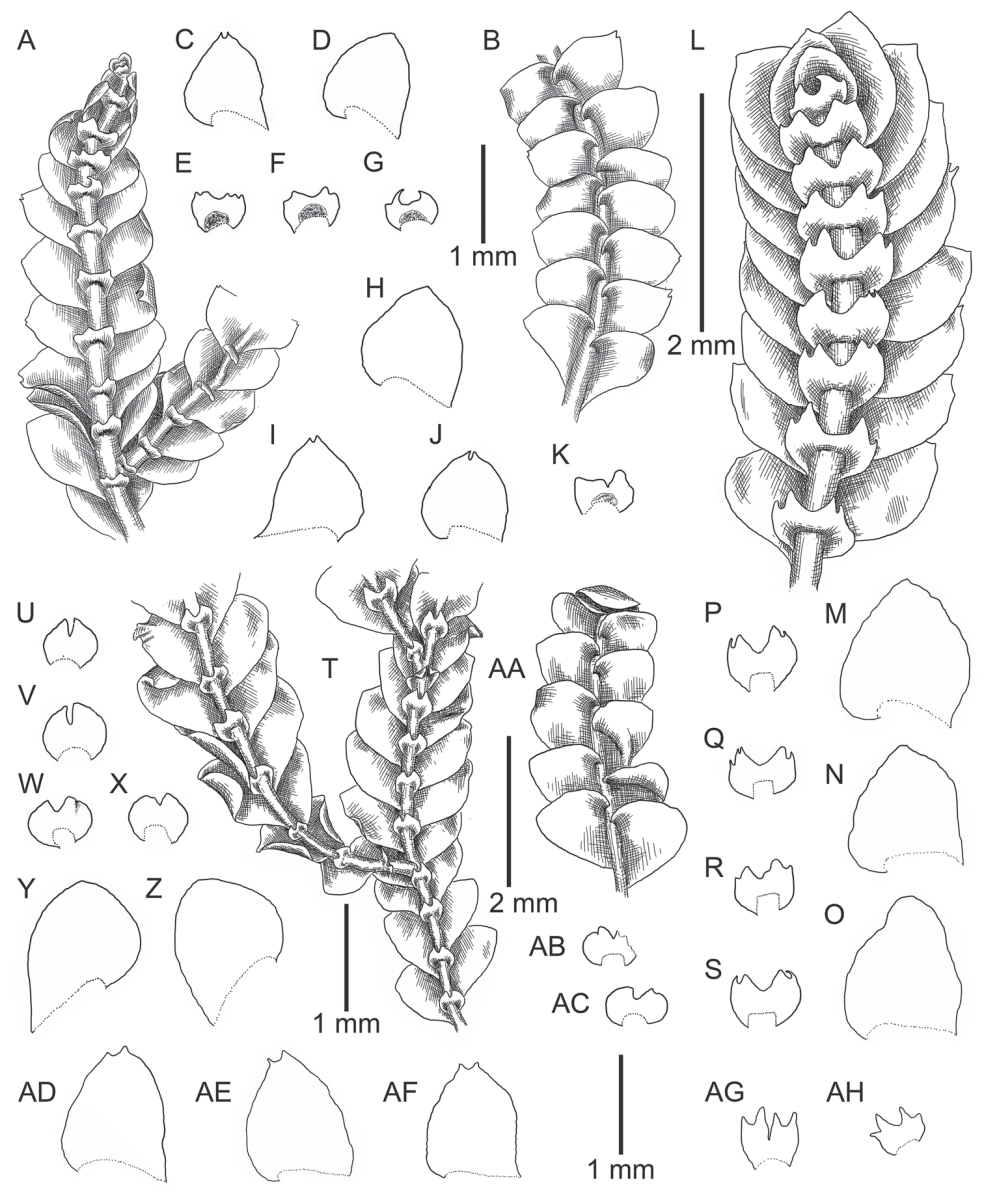
*Calypogeia granulata* Inoue: **A** plant habit, fragment, ventral view **B** plant habit, fragment, dorsal view **C, D, H, I, J** leaves **E, F, G, K** underleaves *Calypogeia lunata* Mitt.: **L** plant habit, fragment, ventral view **M, N, O** leaves **P, Q, R, S** underleaves *Calypogeia marginella* Mitt.: **T** plant habit, fragment, ventral view **U, V, W, X, AB, AC** underleaves **Y, Z** leaves **AA** plant habit, fragment, dorsal view *Calypogeia tosana* (Steph.) Steph. **AD, AE, AF** leaves **AG, AH** underleaves. **A–G** from Syntype G00114896; **L, P–S** from Long no 10664, JE **H–K** from 18004 TNS 174361; **M–O** from Syntype G00064229/5288; **T–Z** from Syntype G00113555/5289; **AA, AB, AC** from Syntype JE-04005904; **AD, AE, AF, AG, AH** from Lectotype G00047274/26013.

Morphologically, the taxon is similar to *Calypogeia tosana* (due to bisbifid underleaves and acute, sometimes incised leaves), from which it differs in underleaves decurrent for 2/3–3/3 of stem width and oil bodies indicated even in the original label as grayish blue “with numerous granules” (= finely granulate).

The description based on type specimens is as follows: plants green, strongly glistening, translucent, 1.5–2.1 mm wide, 1–3 cm long; stem greenish, soft, 220–320 µm wide, sparsely ventrally branched; rhizoids sparse to common, in unclear loose fascicles, obliquely spreading, grayish; leaves contiguous to subimbricate (overlap 1/3 of above situated leaf), very obliquely to subhorizontally inserted, slightly convex, apical third turned to ventral side, not or shortly decurrent, when flattened -– obliquely triangular-ovate, 900–1000 × 900–1000 µm, very shortly incised or apex apiculate; underleaves obliquely spreading, decurrent for 1/3–2/3 of leaf length, commonly bisbifid, divided by U-shaped sinus, undivided area 2–3 cell high, 250–300 × 550 µm, 1.1–1.6 as wide as stem; cells in the midleaf thin-walled, with vestigial trigones, 32.5–52.5 × 30.0–37.5 µm, cuticle smooth.

**Figure 5.**
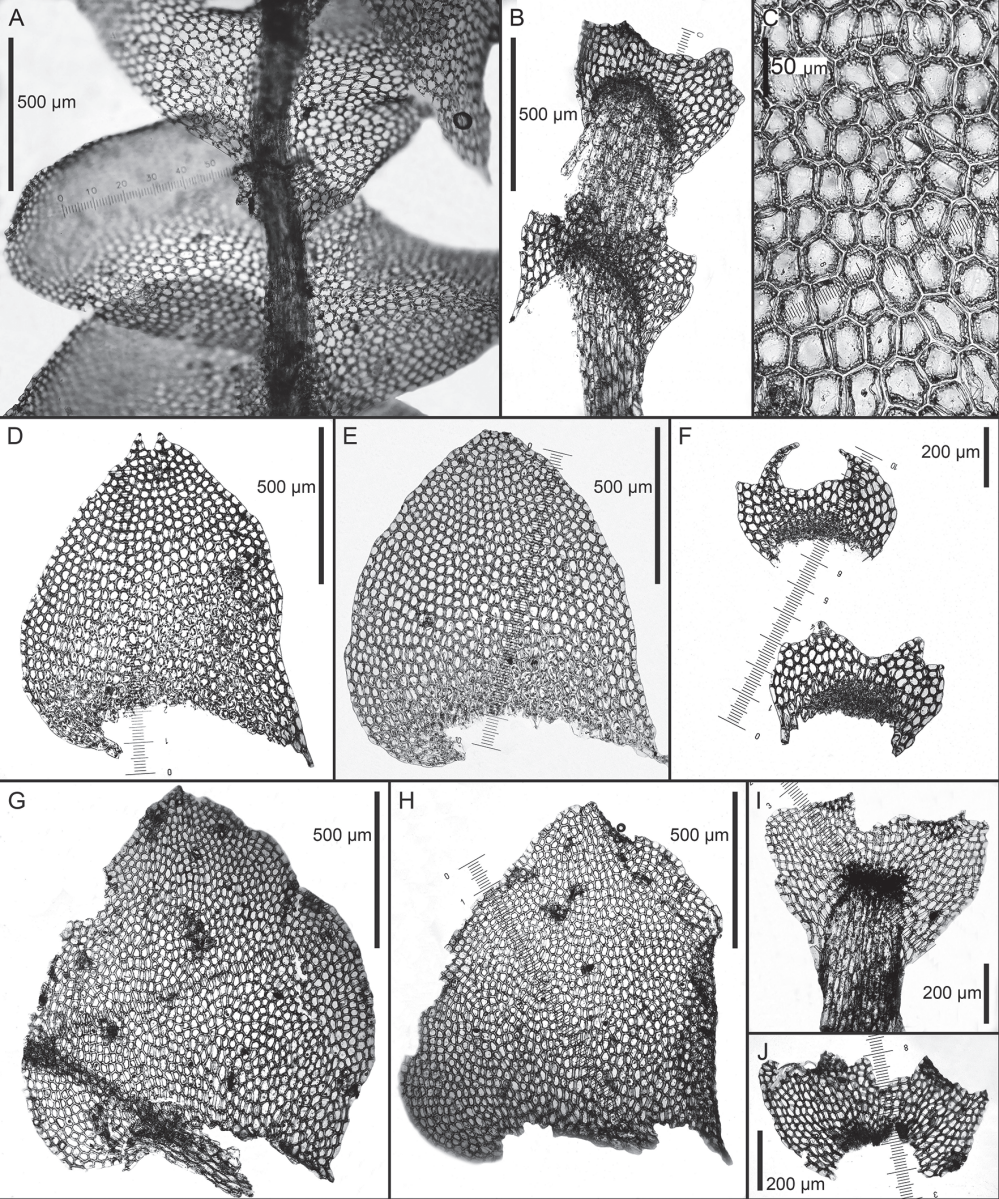
*Calypogeia granulata* Inoue: **A** plant habit, fragment, ventral view **B, F** underleaves **C** leaf middle cells **D, E** leaves *Calypogeia lunata* Mitt.: **G, H** leaves **I, J** underleaves. Scale bars: 500 µm (**A, B, D, E, G, H**); 200 µm (**F, I, J**); 50 µm (**C**). **A, B, C** from Holotype 18004 TNS 174361; **D, E, F** from Isotype G00114896; **G, H, I, J** Syntype G00064229/5288.

#### Calypogeia japonica

Taxon classificationPlantaeJungermannialesCalypogeiaceae

Steph., Sp. Hepat. (Steph.) 6: 448. 1924.

E9808F16-AE42-535F-809A-4E55259E5B15

**Figure 6.**
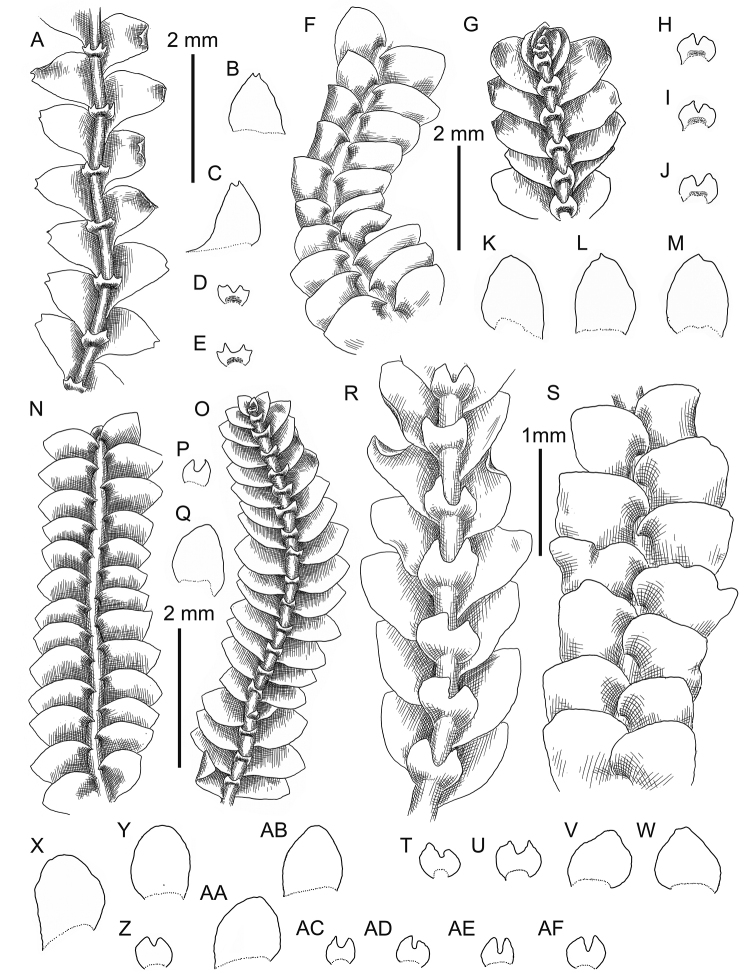
*Calypogeia goebelii* (Schiffn.) Steph.: **A** plant habit, fragment, ventral view **B, C** leaves **D, E** Underleaves *Calypogeia ceylanica* S.Hatt. et Mizut.: **F** plant habit, fragment, dorsal view **G** plant habit, fragment, ventral view **H, I, J** underleaves **K, L, M** leaves *Calypogeia cuspidata* (Steph.) Steph.: **N** plant habit, fragment, dorsal view **O** plant habit, fragment, ventral view **P** underleaf **Q** leaf *Calypogeia japonica* Steph. **R** plant habit, fragment, ventral view **S** plant habit, fragment, dorsal view **T, U, Z, AC, AD, AE, AF** underleaves **V, W, X, Y, AA, AB** leaves. **A–E** from Syntype G00115804; **F–M** isotype G00064248; **N–Q** lectotype G00069713; **R–W** from Neotype G00047413/9720; **X–Z, AE, AF** from G00047412/9717; **AA–AD** from holotype of *Calypogeia ovifolia* H. Inoue TNS76048.

Figures 6R–Z, AA–AF, 7J–L

= Calypogeia ovifolia Inoue Mem. Natl. Sci. Mus. (Tokyo) 16: 100. f. 1: 1–2, 2. 1983.Type: Japan. Between Ashi-kosen and Mt. Torihana, Asahi Mts., Yamagata Pref., ~600 m, H. Inoue, no. 32885 (holotype TNS [TNS76048!]).

##### Type.

Japan. “Japonia, Uematsu” (neotype by Furuki and Ota (2001): G [G00047413/9720!]).

##### Remarks.

For a long time regarded as a Japanese endemic species, it was later reported from Fujian (Zhu et al. 2002, as *C. tsukushiensis* Amakawa) and Guizhou (Bakalin et al. 2015) provinces of China, the Korean Peninsula (Choi et al. 2011) and the southern Kurils (Bakalin et al. 2019c). The distinctive features of the species are biconcentric oil bodies in midleaf cells, deeply divided, not decurrent underleaves (similar to that in *C. neogaea* (R.M. Schust.) Bakalin) and rounded leaves (similar to that in *C. integristipula*). Dry plants may be likely mistaken for *C. muelleriana* with which, however, the distribution area may overlap in the southern Kurils only.

*Calypogeia japonica* was described by Stephani (1924) based on a specimen collected by U. Faurie. However, the collection in G contains the only specimen collected by Faurie (G00047412/9717!), which is from Quelpart Island (= Jeju-do, Korea). Since Stephani sometimes treated Quelpart Island as part of mainland Japan (this was the reason for the geographic mistakes), this specimen might be regarded as a holotype. However, we agree with Furuki and Ota’s (2001) neotypification since the neotype contains much better developed plants and is larger than the specimen from Korea, although the specimen from Japan (and two more, preserved in G) was collected by E. Uematsu, not by Faurie. Moreover, even Stephani annotated the specimen from Quelpart as ‘spec. pessimum’; it would appear strange to regard this as of this type since other specimens in his herbarium provide more copious material.

The description based on the neotype is as follows: plants 1.8–2.0 mm wide, yellowish brownish, merely soft, loosely translucent; stem 220–280 µm wide, sparsely ventrally branched rhizoids common, but not numerous, in loose, obliquely to erect spreading fascicles or separated; leaves obliquely inserted and oriented, slightly concave-canaliculate, contiguous, to slightly overlapping above situated leaves, somewhat loosely crispate along margin, widely ovate-triangular, apex obtuse to narrowly rounded, 1000–1130 × 1000–1250 µm; underleaves obliquely spreading, decurrent for 1/3–2/3 of stem width, divided by V- to U-shaped sinus into two lobes without additional lateral teeth, undivided zone 4–6 cells high, 2.5–3.0 as wide as stem; midleaf cells 25–55 × 25–35 µm, thin-walled, trigones small to very small, cuticle smooth.

**Figure 7.**
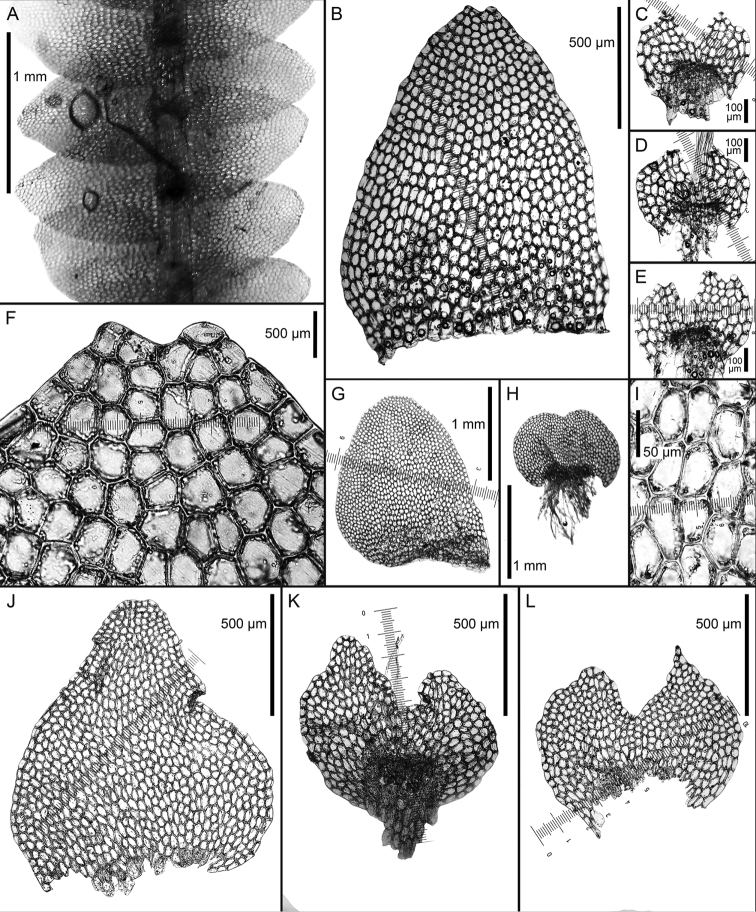
*Calypogeia cuspidata* (Steph.) Steph.: **A** plant habit, fragment, ventral view **B** leaf **C, D, E** underleaves **F** leaf apex cells *Calypogeia integristipula* Steph.: **G** leaf **H** underleaf **I** leaf middle cells *Calypogeia japonica* Steph. **J** leaf **K, L** underleaves. Scale bars: 1 mm (**A, G, H**); 500 µm (**B, F, J, L, K**); 100 µm (**C, D, E**); 50 µm (**I**). **A, B, C, D, E, F** from Lectotype of *C. hawaica* G00067698; **G, H, I** from Lectotype G00061108/26879; **J, K, L** Neotype G00047413/9720.

#### Calypogeia lunata

Taxon classificationPlantaeJungermannialesCalypogeiaceae

Mitt., J. Proc. Linn. Soc., Bot. 5 (18): 107. 1860 [1861].

4BA5F3F3-7C73-5C2F-9187-AB917F6F7429

Figures 4L–S , 5G–J

##### Type.

India. Assam: Griffith (syntype: G [G00064229/5288!]).

##### Remarks.

This is a broadly Sino-Himalayan endemic species that seems locally abundant in the eastern Sino-Himalaya. Mitten (1860) described *Calypogeia lunata* from Assam; later, Singh and Nath (2007a) recorded it from the East Khasi Hills and West Khasi Hills in India. Aside from India, the species was reported from eastern Nepal, Bhutan, Thailand and Yunnan Province in China (Mizutani 1979; Lai et al. 2008; Kitagawa 1988; Hattori 1975, Piippo 1990; Piippo et al. 1998; Long and Grolle 1990). The origin of the report of the species for Yunnan is unclear. Piippo (1990) mentioned *C. lunata* for Yunnan with reference to Grolle (1966), who does not, however, provide label data for this species in Yunnan, although it is also indicated in the review of the general distribution.

The description based on the isotype is as follows: plants brownish to blackish brownish in the herbarium, translucent, glistening, 1.5–2.2 mm width; stem 120–220 µm wide, branching not seen; rhizoids sparse to common in brownish grayish, erect to obliquely spreading loose fascicles; leaves overlapping ~1/4–1/3 of next leaf basal part, slightly convex, with apices somewhat turned to ventral side, obliquely inserted and oriented, ventrally clearly decurrent to 1.0 of stem width or less, widely triangular-ovate, apex acute to obtuse (very rarely bilobed), 850–1200 × 770–1200 µm, margin entire to somewhat crispate; underleaves decurrent for (0.3–)0.5–1.0 of stem width, 1.5–3.5 as wide as stem, bisbifid or with each main lobe divided into three small lobes, or bisbifid with additional lateral tooth on each side; midleaf cells thin-walled, trigones vestigial, cuticle smooth, 30–38 × 20–33 µm (the cell measurements may be incorrect because of collapsed leaf cells).

The species is most morphologically similar to *Calypogeia goebelii* (Kitagawa 1988), from which, however, it differs in underleaf width and shape, long decurrency of underleaves, rarely shortly bifid leaves (versus underleaves commonly less than 2 times as wide as stem and leaves deeply incised). In contrast to mainly Malesian-Papuasian *C. goebelii*, *C. lunata* is characterized by an eastern Sino-Himalayan distribution, where *C. goebelii* can hardly be expected. We hypothesize that the reports of *C. goebelii* from Thailand (Kitagawa 1988 and subsequent mentions based on this) represent the ill-developed modification (probably from dry habitats) of *C. lunata*. On the other hand, *C. lunata* seems to be very closely morphologically related to *C. latissima* (Philippines, see below), from which, however, it differs in its completely smooth cuticle and very rarely (as exclusion) bidentate leaves.

One more observation should be made on the type specimen identification. The specimen in JE marked as the possible type (JE-H2316 = JE04005930!) is actually not the type. The label means that the specimen was collected in “Khasia, Churra”, not in Upper Assam, as in the original description by Mitten (1860). The plants in the Jena ‘type’ are different from the typical *C. lunata* and rather resemble *C. tosana* or *C. goebelii*, although they differ from both in thickened leaf cell walls in dorsal half of leaves (especially in the external wall), V-shaped leaf sinus and 3–5 cells high undivided portion of underleaf. We speculate that the specimen may belong to an undescribed taxon, but we refrain from describing it here until fresh material suitable for DNA and oil body characteristics is obtained.

#### Calypogeia sinensis

Taxon classificationPlantaeJungermannialesCalypogeiaceae

Bakalin & Buczk. PLoS ONE 13(10): e0204561 [13]. 2018.

3A3EAF92-A29F-53C8-8F0B-81378E599177

##### Type.

China. Guizhou Province: Duyun Municipality (26°22.383'N, 107°21.35'E), 1300 m alt., 22 Nov 2013, V.A. Bakalin China-56-77-13 (holotype: VBGI!; isotype: POZW!).

##### Remarks.

The species was described in Guizhou Province, China, and confirmed in northern Vietnam (Buczkowska et al. 2018) but seems hardly restricted by known localities and is likely much more widely distributed. We (Buczkowska et al. 2018) expected its occurrence in the Meta-Himalaya, as well as in Hengduan, which is the area of occurrence of several Sino-Himalayan species of the group to which the present taxon should belong. Some of the records of *Calypogeia azurea* probably belong to this taxon (see doubtful records). The description and illustrations of the taxon were published recently, and no additional information seems required here.

#### Calypogeia tosana

Taxon classificationPlantaeJungermannialesCalypogeiaceae

(Steph.) Steph., Bull. Herb. Boissier (sér. 2) 8 (9): 678 (410). 1908.

5A06EBAF-CBF0-58DB-AA88-FC0A7D67A6FD

= Calypogeia granditexta Steph. Species Hepaticarum 6: 448. 1924. Syn. nov. Type: Japan “Sendai” Uematsu 23 November 1907 (LECTOTYPE (designated here): G G00283130!; another syntype, G00283028!, contains rather typical C. orientalis).

##### Basionym.

*Kantius tosanus* Steph., Hedwigia 34 (2): 54, 1895.

##### Type.

Japan: Tosa Makino (LECTOTYPE (designated here): G [G00047274/26013, packet b!] The holotype should be in ‘herb. Polytechnicum Zurich’, but such specimen is absent in Zurich herbaria (https://www.herbarien.uzh.ch/en/belegsuche.html), therefore we were obliged to lectotypify the species by the specimen from G).

##### Remarks.

This is a widely amphi-Pacific East Asian species whose area stretches from the southern Kurils and East Manchurian mountains in Russia via the Korean Peninsula and Japanese Archipelago to southeastern China, namely, Taiwan (Wang et al. 2011), Guangxi (Zhu and So 2003), Hong Kong (Zhang and Lin 1997), Anhui, Jiangsu, Guangdong, Hainan (Piippo 1990), and Guizhou (Bakalin et al. 2015; Buczkowska et al. 2018) and southward to northern Vietnam (Shu et al. 2017; Bakalin et al. 2018). This is one of the most common species in amphi-Pacific East Asia; however, it hardly penetrates into the Asian mainland. Admittedly, this species is quite morphologically polymorphous, although its polymorphism has probably been somewhat overestimated. Iwatsuki (2001) provides the key to *Calypogeia* in Japan, where the ‘races’(?) with both verruculose cuticle and smooth cuticle are identified as the single *C. tosana*. We hypothesize that these two ‘races’ may represent two different species. The type of *C. tosana* is characterized by a smooth leaf cuticle; this feature, although not mentioned in the original description under *Kantius tosanus* (Stephani 1895), was provided later when a new combination under *Calypogeia* was created (Stephani 1908). The concept of *C. tosana* is here accepted in the narrow sense closely following the type.

There is a problem with the type of plants in the type specimen due to mixture within. The type specimen (Makino 25, G), as correctly noted by T. Furuki *in litt*., contains two intermixed species, with one belonging to true *C. tosana* (coinciding with the original description, packet b) and the other probably belonging to an undescribed taxon. We prefer not to describe this taxon here (it is also beyond the scope of the present account) since the re-collection of fresh material and the study of the ‘intravitam’ character of the taxon (oil body characteristics) and DNA sequences should provide a much better understanding of the taxonomic position of the taxon than the study of poorly preserved sterile and old material in Stephani’s herbarium.

The brief description based on the plants belonging to *Calypogeia tosana* is as follows: plants translucent, glistening, brownish; leaves very shortly bilobed by U-shaped sinus; underleaves uniformly bisbifid (both small and larger) with undivided portion 1–3 cells high, cells in the midleaf thin-walled with small and concave trigones, 30–50 × 22–45 µm and smooth cuticle.

The plants in *Calypogeia granditexta* in G00283130 (lectotype) are very similar in their relatively narrow, shortly decurrent, uniformly bisbifid underleaves, shortly bifid leaves and smooth cuticle to *C. tosana*, and no differences of the species rank were found. Before (Inoue 1974) *C. granditexta* was regarded as the synonym of *C. angusta*, from which, however, differs in incised (versus rounded) leaves and deeply bisbifid (versus bifid, although sometimes with additional lateral teeth in each side) underleaves. Calypogeia granditexta var. anisophylla S. Hatt Journal of Japanese Botany 20: 262. f. 45. isotype (Japan, “Fukushima County, Oze”, 1500 m a.s.l. 7 July 1941 S. Hattori, 451 (G00112334!)) contains fairly typical *Calypogeia integristipula* Steph. and has nothing to do with *Calypogeia tosana*. The holotype of var.anisophylla was not studied physically, although the photographs provided at TNS herbarium site (http://www.type.kahaku.go.jp/TypeDB/bryophyta/41) correspond well to *C. tosana*, but not to *C. angusta*, neither to *C. integristipula*.

**Figure 8.**
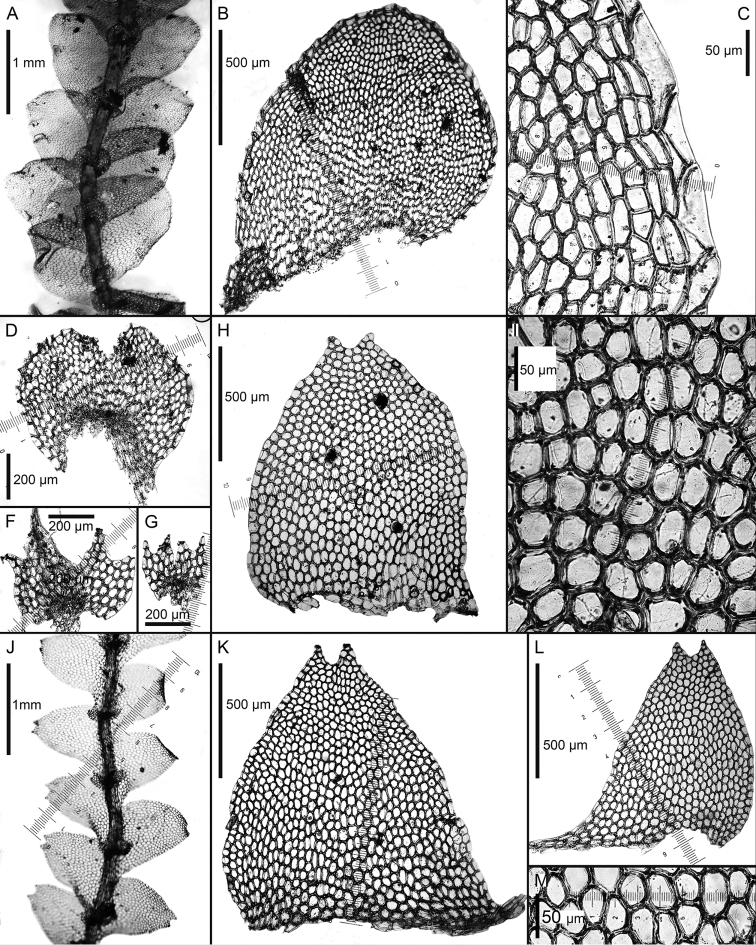
*Calypogeia marginella* Mitt.: **A** plant habit, fragment, ventral view **B** leaf **C** leaf margin cells **D** underleaf *Calypogeia tosana* (Steph.) Steph.: **F, G** underleaves **H** leaf **I** leaf middle cells *Calypogeia goebelii* (Schiffn.) Steph. **J** plant habit, fragment, ventral view **K, L** leaf **M** leaf middle cells Scale: 1 mm (**A, J**); 500 µm (**B, H, K, L**); 200 µm (**D, F, G**); 50 µm (**C, I, M**). **A, B, C, D** from Syntype G00113555/5289; **F, G, H, I** from Holotype G00047274/26013; **J, K, L, M** Syntype G00115804.

#### Calypogeia vietnamica

Taxon classificationPlantaeJungermannialesCalypogeiaceae

Bakalin et Vilnet Herzogia 32 (1): 225. 2019.

5171563A-45A4-50C9-B780-C318A26A4FE3

##### Type.

Vietnam. Lao Cai Province: SaPa District, San Sa Ho Commune, Hoang Lien Range, Phan Xi Pang Peak area, *Rhododendron*-dominated forest with bamboo thickets and many rocky outcrops, moist cliffs in partial shade (22°18.45'N, 103°46.567'E), 2900 m alt., 20 April 2017, V.A. Bakalin & K.G. Klimova V-9-23-17 (holotype: VBGI!).

##### Remarks.

This taxon was recently described in moist rocky outcrops at the highest elevation in Indochina – Phan Xi Pang Mt. – in the somewhat unique formation of a ‘mossy’ *Rhododendron* forest resembling mossy forests occurring in humid tropics, although different from the latter florogenetically (cf. Bakalin et al. 2019b). The species is characterized by blue oil bodies and noticeably large underleaves (only slightly smaller than the leaves) divided by a U-shaped sinus descending to 2/5–1/2 of underleaf length. The species may be expected in other areas of mountainous Indochina, if not spread more widely to the eastern Sino-Himalaya. The description and illustrations were published very recently, and it seems that no more information should be added here.

### Doubtful records

#### Calypogeia azurea

Taxon classificationPlantaeJungermannialesCalypogeiaceae

Stotler et Crotz, Taxon 32 (1): 74. 1983.

D0267F03-93BE-5EB6-B646-D40E3FBA6A9A

##### Type.

Not seen.

##### Remarks.

There are several records of this taxon in East and Southeast Asia. Singh and Nath (2007a) recorded it for the East Khasi Hills; Shu et al. (2017) reported it for northern Vietnam; Wang et al. (2011) mentioned it for Taiwan based on two records of ‘*Calypogeia trichomanis*’. Zhu and So (2003) recorded taxon for Guangxi Province. As shown by Buczkowska et al. (2018), the traditionally named *C. azurea* should be subdivided into at least three main lineages: ‘true’ *C. azurea* in Europe, a North American semicryptic and still validly not described taxon, and the taxon morphologically similar to European *C. azurea* but distributed in East Asia that was described as *C. orientalis* in l.c. Geography-correlated infraspecific variability was also observed within *C. orientalis*; two subspecies may be maintained, both of which are distributed in temperate zone, with one restricted to continental mainland (Korean Peninsula, Russian Manchuria) and the other occurring in Japan. The occurrence of *C. orientalis* was not confirmed in China, although it is highly probable in the northeastern part of the country. Due to data in hand, *C. orientalis* is only known from Russian Manchuria, Sakhalin and Kuril Islands, Japan and the Korean Peninsula, being most common between 35 and 45°N (in cool temperate to hemiboreal zones). Thus, some records of *Calypogeia azurea* in Northeast China may actually belong to *C. orientalis*, but specimens from the Sino-Himalaya could hardly belong to this species. Another recently described taxon, *C. sinensis*, with exceedingly deep blue oil bodies (described based on material from northern Vietnam and Guizhou Province in China, where both specimens were preliminarily named *C. azurea*) may be the taxon previously misidentified as *C. azurea* in the aforementioned works. Bapna and Kachroo (2000) reported occurrences of *C. trichomanis* in Darjeeling and Nepal; what these reports mean is difficult to say, but some of them probably also belong to *C. sinensis*.

#### Calypogeia fissa

Taxon classificationPlantaeJungermannialesCalypogeiaceae

(L.) Raddi, Jungermanniogr. Etrusca: 33. 1818.

3E7FF876-3457-5F96-A97E-582818D932EF

##### Basionym.

*Mnium fissum* L., Sp. Pl. 1: 1114. 1753. nom. conserv. Original material: Great Britain, Surrey, Dorking; not seen.

##### Remarks.

*Calypogeia fissa* is one of the oldest names in *Calypogeia*, and several taxa were split from the original *C. fissa* s.l. The species seems to be restricted to Europe. Within North America and the northwestern amphi-Pacific (Commanders, Kamchatka, Kurils, Sakhalin), *C. fissa* is substituted by *C. neogaea* (R.M. Schust.) Bakalin. Stotler and Crandall-Stotler (2017: 591) noted that *C. fissa* “likely does not occur in North America and specimens identified as such likely belong to *C. neogaea*”. In an older time, *C. fissa* was recorded in Japan, although it was doubted as early as Hattori (1952) and then was never mentioned for the Japanese flora. The nearest morphological ally of *C. fissa* in temperate East Asia is *C. tosana*.

Nevertheless, *Calypogeia fissa* was several times recorded even at a relatively recent time for the East Asian mainland: Singh and Nath (2007a) recorded it for the East Khasi Hills and West Khasi Hills as well as (presumably based on other literature records, unfortunately not cited in l.c.) for Sikkim and Darjeeling. Bapna and Kachroo (2000) described its wide distribution in India. Wang et al. (2011) mentioned it for Taiwan; Fang et al. (1998), for Jiangxi. The records of the species for Yunnan and Hunan are based on Nicholson et al. (1930). Presumably, the vast majority of records of *C. fissa* may be based on misidentifications of *C. tosana* (if so, the latter is much more widely distributed on the Asian mainland than would be obvious if only available publications were taken into account). We hypothesize that ‘true’ *Calypogeia fissa* should be restricted to Europe from where the only accessions were confirmed by Buczkowska et al. (2018), and that the species should be excluded from the Sino-Himalayan *Calypogeia* flora. Moreover, even in Europe, *Calypogeia fissa* is represented by two genetically well-separated taxa (Buczkowska et al. 2011) that probably require taxonomic revision.

#### Calypogeia goebelii

Taxon classificationPlantaeJungermannialesCalypogeiaceae

(Schiffn.) Steph., Bull. Herb. Boissier (sér. 2) 8 (9): 677 (409). 1908.

E4CBED16-84BE-5019-A64C-D63F5B76BC55

Figures 6A–E , 8J–M

##### Basionym.

*Kantius goebelii* Schiffn., Nova Acta Acad. Caes. Leop.-Carol. German. Nat. Cur. 60 (2): 260. 1893.

##### Type.

Java. K. Goebel (syntype: G [G00115804!]).

##### Remarks.

The species was described from Java based on K. Goebel specimen (Schiffner 1893) and is mostly Malesian-Papuasian in distribution, probably reaching westward to northern Thailand (if the report by Kitagawa 1988 is correct) and spreading eastward to Samoa. We did not see the specimens of this species from northern Indochina. However, *Calypogeia lunata* is quite abundant, morphologically malleable and provides some modifications superficially resembling *C. goebelii* in northern Vietnam, although never having such distinctly lobed leaves as occur in ‘true’ *C. goebelii*, nor narrow underleaves (1.5–2.0 as wide as the stem, as commonly occurs in *C. goebelii*). Moreover, Kitagawa (1988) did not observe blue oil bodies in his specimens, and he provides a yellowish color for the plants, whereas the plants that have blue oil bodies commonly develop greenish-whitish to grayish pigmentation in the herbarium. Thus, it is an open question whether the specimens named *C. goebelii* by Kitagawa truly even belong to the blue-oil-bodied *Calypogeia* complex. The type of *C. goebelii* is actually similar to that of *C. tosana* in general outlook, and the differentiation from the latter in the absence of oil bodies is quite troublesome. Therefore, we are unable to confirm or reject this species from the northern Indochinese flora, although we doubt it.

The description based on the isotype is as follows: plants brownish, pellucid, glistening, 1.5–2.5 mm wide, 5–8 cm long; stem 150–200 µm wide, sparsely ventrally branched; rhizoids brownish, common to numerous, obliquely to erect spreading fascicles; leaves contiguous to somewhat distant, slightly convex, decurrent for 1–2 stem widths, 750–1250 × 575–1050 µm, divided by U-shaped sinus into two acute lobes; underleaves, obliquely spreading, 1.5–2.5 as wide as stem, bisbifid, the undivided portion in the underleaf middle 2 cells high, arcuately inserted, not or barely decurrent; midleaf cells thin-walled, trigones very small, concave, 37.5–55.0 × 25.0–37.5 µm.

#### Calypogeia muelleriana

Taxon classificationPlantaeJungermannialesCalypogeiaceae

(Schiffn.) Müll.Frib., Beih. Bot. Centralbl. 10 (4/5): 217. 1901.

3306A20E-109A-516A-83DA-8FF91E5491C9

##### Basionym.

*Kantius muellerianus* Schiffn., Sitzungsber. deutsch. naturwiss.-med. Vereins Böhmen “Lotos” Prag 48: 342. 1900.

##### Original material.

Czech Republic, Bohemia, Schiffner; not seen.

##### Remarks.

This boreal species was originally described from the border between the Czech Republic and German Bavaria (Bohemian Forest) and was found to have circumpolar distribution in the hemiarctic and boreal zones of the Northern Hemisphere. However, even in the hemiboreal zone of East Asia (e.g., in the southern Russian Far East at 43–48°N), the species rarely occurs. *Calypogeia muelleriana* seems to be hardly possible even in Northeast China, as mentioned by Piippo (1990). Two recent reports from Guangxi and Jiangxi Provinces of China (Zhu and So 2003; Fang et al. 1998) may belong to other taxa, such as *C. apiculata*, *C. sinensis*, and *C. granulata*, whose distribution in the Sino-Himalaya is underestimated. The distinct differentiation features of *C. muelleriana* are highly undivided underleaf lamina (4–5 and more cells high), rounded to rarely obtuse leaf apices and grayish to colorless botryoidal oil bodies. The European materials of *Calypogeia muelleriana* are split into two different and perhaps cryptic taxa (Buczkowska 2010, Buczkowska and Bączkiewicz 2011).

#### Calypogeia neesiana

Taxon classificationPlantaeJungermannialesCalypogeiaceae

(C. Massal. et Carestia) Müll.Frib., Verh. Bot. Vereins Prov. Brandenburg 47: 320. 1905.

49CA330F-846F-5E69-8C1F-41DB5549A05A

##### Basionym.

Kantius trichomanis var. neesianus C. Massal. et Carestia, Nuovo Giorn. Bot. Ital. 12 (4): 351. 1880.

##### Original material.

Italia, Rive Valsesia; not seen.

##### Remarks.

The species was described from the Italian Alps (Massalongo and Carestia 1880) and later found as a sub-circumpolar (distinctly more common in amphi-oceanic areas) boreal and mainly montane species. The area of the taxon largely overlaps that of *C. integristipula*, although *C. neesiana* seems to be much rarer than the former, more inclined to inhabit decaying wood and slightly more southern in distribution (reaching to Japan and the Korean Peninsula; in both these sites, it is represented by Calypogeia neesiana subsp. subalpina (Inoue) Inoue). The species likely occurs in Northeast China; however, it has not been recorded there. The reports of the species from the southern half of China (Anhui, Jiangxi, Taiwan, and Yunnan, cf. Wang et al. 2011, Piippo et al. 1997, Fang et al. 1998; Piippo 1990) are at least partly based on *Calypogeia cordistipula* (synonymized with *C. neesiana* by Piippo et al. 1997) – another taxon accepted here with species status. Therefore, we doubt the occurrence of this species in the Sino-Himalaya and expect at least some of these records to be referred to *C. cordistipula*.

#### Calypogeia sphagnicola

Taxon classificationPlantaeJungermannialesCalypogeiaceae

(Arnell et J.Perss.) Warnst. et Loeske, Verh. Bot. Vereins Prov. Brandenburg 47: 320. 1905.

7A48679F-08A0-5D9D-B80F-9CB7F3DD1607

##### Basionym.

*Kantius sphagnicola* Arnell et J.Perss., Rev. Bryol. 29 (2): 26. 1902.

##### Original material.

Sweden, Dalarne; not seen.

##### Remarks.

Buczkowska et al. (2012a, 2012b) showed that *Calypogeia sphagnicola* is a complex of distanced taxa (at least three species should be recognized), where additional study is required to name all revealed entities. To date, this problem has not been resolved, and it is unclear what taxon is recorded for Guangxi Province in China (Zhu and So 2003). Within China, this species is also recorded for Jilin (Piippo 1990), but the specimen may belong to another species.

#### Calypogeia trichomanis

Taxon classificationPlantaeJungermannialesCalypogeiaceae

(L.) Corda Naturalientausch 12 [Opiz, Beitr. Naturgesch.] : 653. 1829. Rejected name, Art. 56, Szenzhen Code

4F8C3C75-3805-5531-94D5-BCC16859DDB0

##### Remarks.

There are several records of this rejected name. *Calypogeia trichomanis* was treated very broadly in former times. Mitten (1860) reported it from the Sikkim and Khasia Mountains, and Noguchi et al. (1966) recorded it for eastern Nepal. Piippo (1990) indicated it for Jilin, Anhui and Taiwan in China. Bapna and Kachroo (2000) reported several occurrences in India. In Europe, this species was sometimes estimated as the current *Calypogeia azurea* taxon, which is not present in East Asia (Buczkowska et al. 2018). The understanding of “*C. trichomanis*” in East Asia is additionally complicated by synonymization of other names with ‘*C. trichomanis*’; e.g., Hattori (1952) synonymized *C. angusta* under *C. trichomanis* and created additional confusion.

### Taxa that are not recorded but may be expected

#### Calypogeia asakawana

Taxon classificationPlantaeJungermannialesCalypogeiaceae

S. Hatt. ex Inoue, J. Jap. Bot. 39 (4): 107. 1964.

B5C02B09-C85D-5B47-9681-6ECE1F494680

Figure 9A–D

= Calypogeia okamurana Steph. ex Bonner, Index Hepaticarum 3: 501, 1963 (nom. inval., Art. 38.1(a), no description). Authentic material: Japan. Iyo: Tokonabe Mt., 30 March 1913, S. Okamura no. 383 (original material, probably scheduled as the type: G [G00067726!], the specimen in all ways is similar to C. asakawana).

##### Type.

Japan. Tokyo: Asakawa Experimental forest, 12 June 1954, U. Mizushima, no. 5 (holotype: TNS [TNS-174359!]; isotype NICH [NICH-55582!]).

##### Remarks.

The species is regarded as Japanese endemic (known in Honshu only, cf. Yamada and Iwatsuki 2006) and is characterized morphologically by small, deeply divided, bifid and spreading underleaves (slightly wider than stem) and rounded leaf apices.

The description based on the holotype is as follows: plants pale brownish in herbarium, prostrate, translucent, slightly glistening, 1.1–1.5 mm wide and 8–20 mm long, forming loose mats; rhizoids rather numerous, originating as several unclear fascicles near underleaf bases and obliquely to erect spreading, attaching plants to the substratum; stem brownish (in the herbarium), 100–160 µm in diameter, branching not seen; leaves subhorizontally inserted, dorsally insertion line subtransverse to loosely arcuate, ventrally decurrent for 1/2–2/3 of stem width, obliquely lingulate, to obliquely ovate-lingulate, slightly convex to planar, slightly undulate along margin, laterally spreading, 650–700 × 400–550 µm, leaf apex rounded; underleaves loosely sinuately to transversely inserted, shortly decurrent (up 1/3 of stem width), 120–170 × 200–220 µm, bilobed by U-shaped sinus descending to 2/3 of leaf length, undivided zone 1–2 cells, lobes in the base 3–5 cells wide, midleaf cells oblong, thin-walled, 32–62 × 20–33 µm, trigones vestigial, cuticle virtually smooth; cells along leaf margin subquadrate to oblong, 20–38 µm, thin-walled, with very small concave trigones, cuticle smooth.

**Figure 9.**
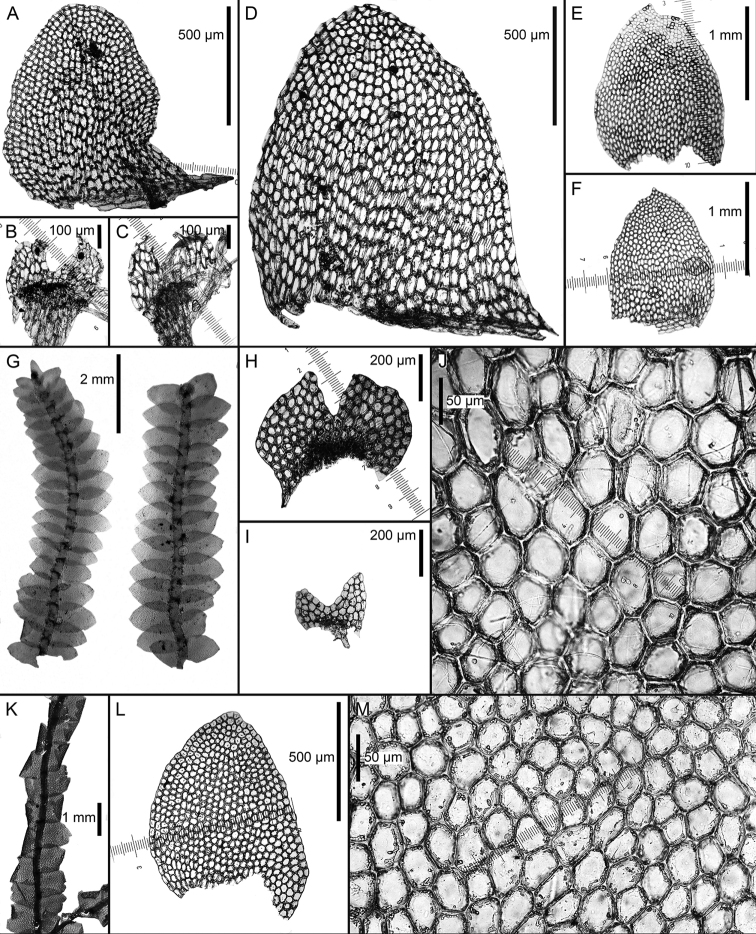
*Calypogeia asakawana* S.Hatt. ex Inoue: **A** plant habit, fragment, ventral view **B, C** underleaves **D** Leaf *Calypogeia ceylanica* S.Hatt. et Mizut.: **E, F** leaves **H** underleaf **J** leaf middle cells *Calypogeia cuspidata* (Steph.) Steph. **G** plant habit, fragment, ventral view **I** underleaf **L** leaf **M** leaf middle cells *Calypogeia decurrens* (Steph.) Steph.: **K** plant habit, fragment, ventral view. Scale bars: 2 mm (**G**); 1 mm (**E, F, K**); 500 µm (**A, D, L**); 200 µm (**H, I**); 100 µm (**B, C**); 50 µm (**J, M**). **A** from Holotype TNS-174359; **B, C, D** from authentic material of *C. okamurana* Steph. nom. herb., G00067726; **E, F, H, J** Isotype G00064248, **G, I, L, M** from Lectotype G00069713; **K** from Isotype G00060745.

#### Calypogeia ceylanica

Taxon classificationPlantaeJungermannialesCalypogeiaceae

S. Hatt. et Mizut., Candollea 23: 288. 1968.

6635E1C3-B7A0-5804-A35A-D727629743A6

Figures 6F–M , 9E–J

##### Type.

Sri Lanka. Central Province: Nuwara-Eliya, 1950 m a.s.l., 24–27 February 1954, F. Schmid 10334 (isotype: G [G00064248!]).

##### Remarks.

*Calypogeia ceylanica* is known as a taxon restricted to Ceylon (Sri Lanka) and was never recorded for the Sino-Himalaya, although it may be expected in Sikkim, Assam, or even farther. Moreover, some reports of *C. muelleriana* may actually be based on *C. ceylanica*. *Calypogeia ceylanica* differs from *C. muelleriana* in more deeply divided and narrower underleaves and apiculate to shortly bidentate leaf apices (a feature that very rarely occurs in *C. muelleriana*).

The description based on isotype plants is as follows: plants yellowish brownish in herbarium, glistening, translucent, 2.2–3.5 mm wide 3–5 cm long; stem 370–450 µm wide, branching not seen; rhizoids in loose colorless to brownish fascicles, sparse to numerous; leaves obliquely inserted, slightly concave or convex, somewhat turned to ventral side, not or barely decurrent ventrally, obliquely ovate, well developed 560–670 × 450–550 µm, apex acute to (rarely) unclearly and very shortly bidentate; underleaves obliquely spreading, 1.1–1.3 as wide as stem, decurrent for ¼–1/3 of stem width, divided by V- to U-shaped sinus into two lobes, lateral teeth absent or present and unclear, undivided portion 2–3 cells high; midleaf cells thin-walled, trigones very small to vestigial, 35–80 × 35–58 µm, cuticle smooth.

#### Calypogeia cuspidata

Taxon classificationPlantaeJungermannialesCalypogeiaceae

(Steph.) Steph., Bull. Herb. Boissier (sér. 2) 8 (9): 669 (401). 1908.

EBC2104F-F4C5-5B5C-8374-FF290975DCD7

Figures 6N–Q , 7A–F , 9G–M

= Calypogeia confertifolia Steph. Species Hepaticarum 6: 447. 1924. Type: Hawaii. 330 m a.s.l. (1000 ft. on the label) (Lectotype (designated here): G [G00067701!] there is no other known authentic materials for this taxon in G).
= Calypogeia hawaica Steph. Bull. Herb. Boissier, sér. 2, 8(9): 663. 1908. Type: Hawaii, Baldwin (Lectotype, designated here: G [G00067698]). The cited specimen should be selected as the lectotype (there are several specimens in the sheet, all collected by Baldwin in Hawaii) because this specimen label bears only measurements handwritten by Stephani. G00282642 contains plants similar to C. tosana (as also annotated by H. Miller) with constantly bifid leaves and bisbifid underleaves. G00282641 is the same as G00282642. G00282640 is the transitional variant between G00282642 and the lectotype. G00282598 is the same as G00282640.

##### Basionym.

*Kantius cuspidatus* Steph., Bull. Herb. Boissier 5 (10): 846. 1897.

##### Type.

Hawaii, Heller 2308 (LECTOTYPE (designated here): G [G00069713!] there are no other known authentic materials for this taxon in G).

##### Remarks.

The species was described from Hawaii and is somewhat morphologically similar to Indochinese-Malesian *C. apiculata*, especially in comparatively small and only shortly decurrent underleaves. It is questionable whether the species may occur in the Sino-Himalaya and Meta-Himalaya, although similar forms, regarded by us as the only forms of *C. apiculata*, were observed in Vietnam. The description from the lectotype of *C. cuspidata* is as follows: plants greenish to brownish greenish, 1.5–2.3 mm wide 2–4 cm long; stem 180–210 µm wide; rhizoids virtually absent or in erect spreading fascicles, rarely occur; leaves contiguous to overlapping for 2/5 of leaf width in the basal part, loosely concave-canaliculate, obliquely ovate, not decurrent, well developed 700–1100 × 550–900 µm, merely acute to obtuse, rarely narrowly rounded; underleaves 1.1–1.4 as wide as stem, arcuately inserted, not or for ¼ of stem width decurrent, divided by U-shaped sinus, undivided portion 2(–3) cells high, lateral teeth absent; midleaf cells thin-walled, trigones very small, concave, 35–53 × 30–40 µm; cuticle smooth.

*Calypogeia cuspidata* differs from *C. apiculata* in not or shortly decurrent underleaves, more densely inserted leaves, wider underleaves with longer lobes, divided by U-shaped sinus and smooth leaf cuticle.

The status of *Calypogeia confertifolia*, synonymized with *C. cuspidata* (Miller et al. 1983, also https://bryophyteportal.org/frullania/taxa/index.php?tid=164252#), is questionable. The description from the lectotype of *C. confertifolia* is as follows: plants greenish brownish, slightly glistening, barely translucent, 1250–2200 µm wide; stem 250–300 µm wide; rhizoids sparse to numerous, in brownish, obliquely to erect spreading fascicles; leaves subimbricate (overlapping to ½ of the next leaf), convex, obliquely inserted and oriented, apical thirds turned to ventral side, not or barely decurrent, obliquely ovate to subrotundate, apex acute to obtuse or rounded, well-developed 800–1000 × 800–1000 µm; underleaves appressed to the stem or very narrowly spreading, 1.5–2.0 as wide as stem, divided by V- to U-shaped sinus into two triangular lobes, without lateral teeth, not decurrent or decurrent to 1/3 of stem width, undivided portion 2–3 cells high; cells in the midleaf thin-walled, 35–55 × 30–45 µm, trigones very small to vestigial, concave; cuticle smooth.

Due to plant features in the type specimen *Calypogeia confertifolia*, it differs from the *C. cuspidata* type in leaf shape, which is convex in *C. confertifolia* but concave-canaliculate in *C. cuspidata*, as well as in wider leaves and thicker stems. Due to limited material available, we still maintain the synonymy of these names.

Another possible synonym of *Calypogeia cuspidata* is *C. hawaica*. The description based on the lectotype is as follows: plants yellowish brownish, merely translucent, more or less soft, 2.0–3.1 mm wide, branching not seen; stem 210–320 µm wide; rhizoids sparse, in some underleaves only, in obliquely spreading brownish fascicles; leaves contiguous to overlapping to 1/3 of the next leaf in the base, nearly planar to very loosely canaliculate-concave, ventrally not decurrent, 800–1400 × 700–1150 µm, obliquely ovate, apiculate, or rarer, apex obtuse or very shortly bidentate (commonly larger leaves); underleaves obliquely spreading, decurrent for 1/3–1/2 of stem width, 1.0–1.2 as wide as stem, divided by V-shaped sinus into two lobes, undivided portion 2–3 cells high, with smooth or without blunt tooth or very shortly bisbifid; cells in the midleaf 37–75 × 37–45 µm, thin-walled, trigones vestigial, cuticle virtually smooth.

*Calypogeia hawaica* may be compared with *C. tosana*, *C. apiculata* and *C. cuspidata*. It is different from *C. apiculata* through its not decurrent leaves and smooth cuticle; from typical *C. cuspidata* in sometimes briefly bifid, narrower and longer decurrent underleaves and sometimes bisbifid leaves; from *C. tosana*, it differs in almost uniformly bifid leaves and bisbifid underleaves (underleaves are wider in *C. tosana*), and more translucent and glistening appearance. The closest morphological relations are to *C. cuspidata*, but this question needs further consideration.

#### Calypogeia decurrens

Taxon classificationPlantaeJungermannialesCalypogeiaceae

(Steph.) Steph., Bull. Herb. Boissier (sér. 2) 8 (9): 675 (407). 1908.

BD538571-9387-55F4-90BC-3788FB18952C

Figure 9K

##### Basionym.

*Kantius decurrens* Steph., Hedwigia 34 (2): 52. 1895.

**Type.** Indonesia, Sumatra, Kehding (isotype: G [G00060745!]).

##### Remarks.

The species status is seriously doubted by Söderström et al. (2016), probably due to supposed close morphological relations to *C. arguta*. However, the taxon is different from *C. arguta* in narrow (not U-shaped, as common in *C. arguta*) leaf sinus and smooth cuticle (versus distinctly papillose) and especially in brown pigmentation of herbarium plants (*C. arguta* is pale even in the very old type in STR). To attract some attention to this very poorly known species (and to stimulate the search for similar forms in the Meta-Himalaya), we include this Indonesian taxon in the key.

#### Calypogeia formosana

Taxon classificationPlantaeJungermannialesCalypogeiaceae

Horik., J. Sci. Hiroshima Univ., Ser. B, Div. 2, Bot. 2: 186. 1934.

EC78791B-5218-518A-BF6A-8790499C04BA

##### Type.

Taiwan (Formosa). Mt. Morrison, August 1932, Y. Horikawa, no. 9124; not seen.

##### Remarks.

This is a Taiwan endemic species (Horikawa 1934) that may be expected in the eastern Meta-Himalaya. The taxon has unclear relationships (placed into “incertae sedis” in Söderström et al. 2016), and by morphology (as it could be estimated from the description and illustration) it is related to *Calypogeia integristipula*, from which, however, it differs in acute leaves. Acute leaves are also similar to many other *Calypogeia* that are recorded or may be expected in the Sino-Himalaya, but all of them have more deeply (more than 1/2) divided underleaves, versus only short and lunate sinus in *C. formosana* underleaves. Another possible morphological relative of *C. formosana* is C. neesiana ssp. subalpina, which is characterized by orbicular and shortly divided underleaves. The two taxa, however, differ in their leaf apex features.

**Calypogeia goebelii var. siamensis N.Kitag., Beih. Nova Hedwigia 90: 165. 1988.**

##### Type.

Thailand. Nakawn Sritamarat: Mt. Khao Luang, M. Tagawa & N. Kitagawa (holotype: KYO [T4737]); not seen.

##### Remarks.

The taxon is known only from the type that is from southern Thailand (Kitagawa 1988) and was never recorded for the Sino-Himalaya. This taxon is indeed different from true *C. goebelii* due to considerably larger leaf cells, more deeply bilobed leaves and fragile apical leaf teeth. This taxon may belong to a species not yet described, but to draw any conclusions, new collections that are suitable for molecular analysis and/or for study of oil body characteristics are needed.

#### Calypogeia integristipula

Taxon classificationPlantaeJungermannialesCalypogeiaceae

Steph., Bull. Herb. Boissier (sér. 2) 8 (9): 662 (394). 1908.

95E1DEDC-BAE1-56C2-AC15-A6172D017518

Figure 7G–I

##### Type.

Germany. Saxonia: July 1888, F. Stephani (lectotype, designated by Bonner (1963) and followed by Grolle (1976): G [G00061108/26879!]).

##### Remarks.

This is a generally boreal circumpolar species widely spreading to hemiarctic and hemiboreal zones and southward in corresponding belts in the mountains (especially in Japan, although surprisingly not known in China and the Korean Peninsula). The description based on the lectotype is as follows: plants 2.2–3.0 mm wide, soft, greenish to yellowish greenish, loosely translucent; stem 200–300 µm wide, freely ventrally branched with 1–2 branches from one underleaf sinus; rhizoids common, in obliquely spreading brownish fascicles; leaves very obliquely inserted, not or barely decurrent ventrally, contiguous to overlapping 1/4 of above situated leaf in the leaf base, slightly convex to nearly planar, ovate to obliquely ovate, 1200–1900 × 1000–1500 µm, with rounded apex; underleaves appressed to the stem, retuse to emarginate at apex, 1.7–2.5 as wide as stem; midleaf cells thin-walled, trigones vestigial, cuticle smooth to very finely verruculose, 37.5–70.5 × 32.5–55.0 µm.

#### Calypogeia khasiana

Taxon classificationPlantaeJungermannialesCalypogeiaceae

Ajit P. Singh et V. Nath, Taiwania 52 (4): 320. 2007.

DC430B2F-ECD2-5774-8659-777C4FA8B494

##### Type.

India. Meghalaya: East Khasi Hills, Langkyrdum-Dawki Road, 07 Nov 1998, V. Nath et al. (holotype: LWG [206109-A]; not seen).

##### Remarks.

Singh and Nath (2007b) described *Calypogeia khasiana* from Khasia Mt. The species is somewhat similar to *C. ceylanica*, which differs in smaller cells and acute (not incised) leaf apex. The differences from *C. lunata* are less clear. Singh and Nath (2007b: 322) noted “*C. lunata* Mitt. differs from *C. khasiana* in having yellow brown color, stem 9–10 cells across and 0.25–0.26 × 0.36–0.38 mm in diameter, leaves obliquely ovate, apex narrowed, obtuse to subacute, bidentate, sinus less broad, acute to obtuse, lobes 2 cells long, underleaves bisbifid, lobes divergent, shallowly and irregularly notched, forming acute-obtuse dentitions”. This list of features is untenable, for instance, because *C. lunata* is not yellowish in the herbarium and has similar (and greatly variable) leaf apex, and the same should be noted about underleaf shape. In our opinion, *C. khasiana* may be only a *C. lunata* habitat modification. The possible difference is in underleaves that are not or barely decurrent in *C. khasiana* (the feature is observed in the picture in the original paper, but no information on this feature is provided in the description), whereas commonly 1/2–1 of stem width decurrent in *C. lunata*. We include it in the key with some doubts, at the same couplets with *C. lunata*.

#### Calypogeia latissima

Taxon classificationPlantaeJungermannialesCalypogeiaceae

Steph., Sp. Hepat. (Stephani) 6: 449. 1924.

E9C3C030-6CA2-5BB1-BFA4-023F9C385B3A

**Figure 10.**
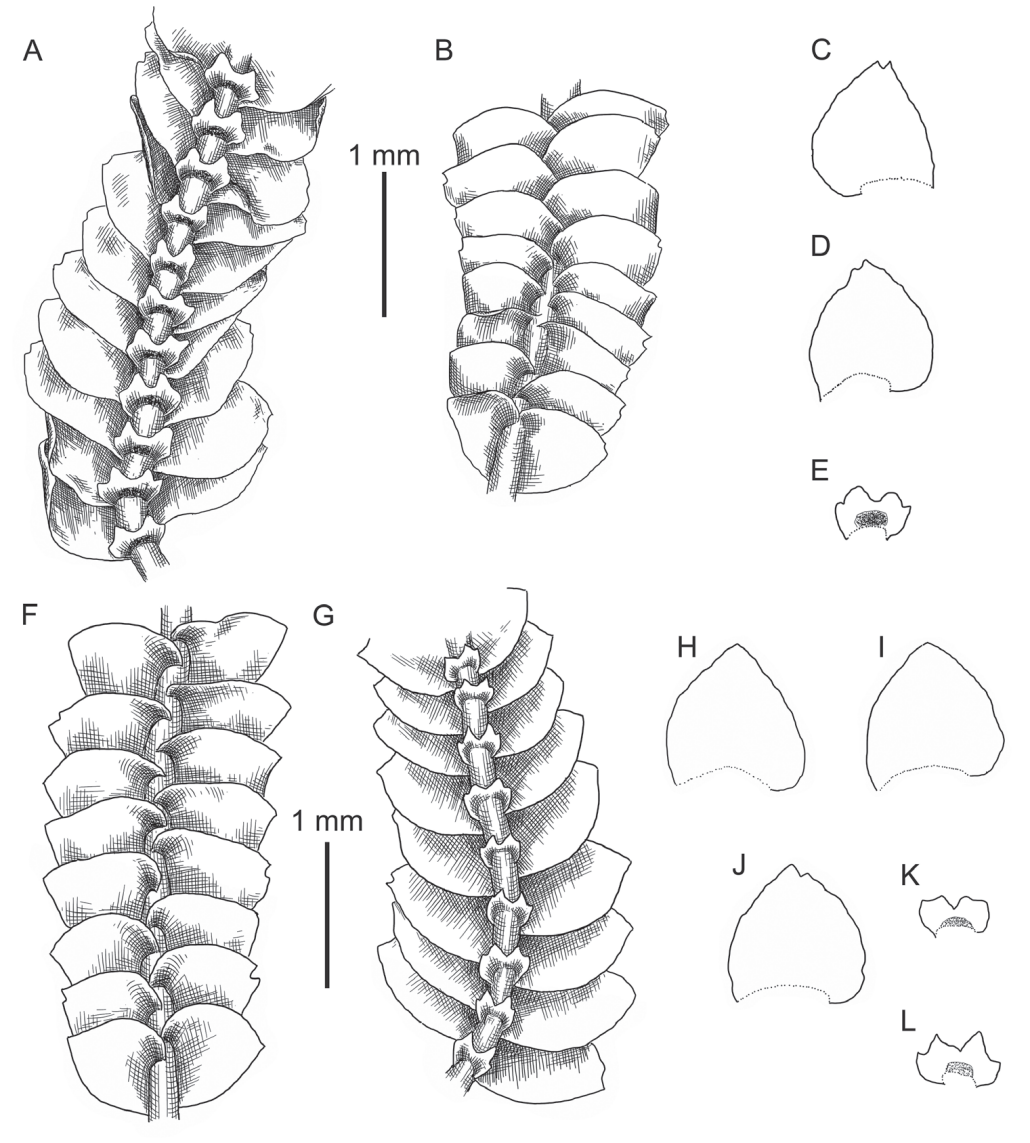
*Calypogeia latissima* Steph. **A** plant habit, fragment, ventral view **B** plant habit, fragment, dorsal view **C, D** leaves **E** underleaf *Calypogeia yoshinagana* Steph. **F** plant habit, fragment, dorsal view **G** plant habit, fragment, ventral view **H, I, J** leaves **K, L** underleaves. **A–E** from Lectotype G00061102; **F–L** Lectotype G00067733.

**Figure 11.**
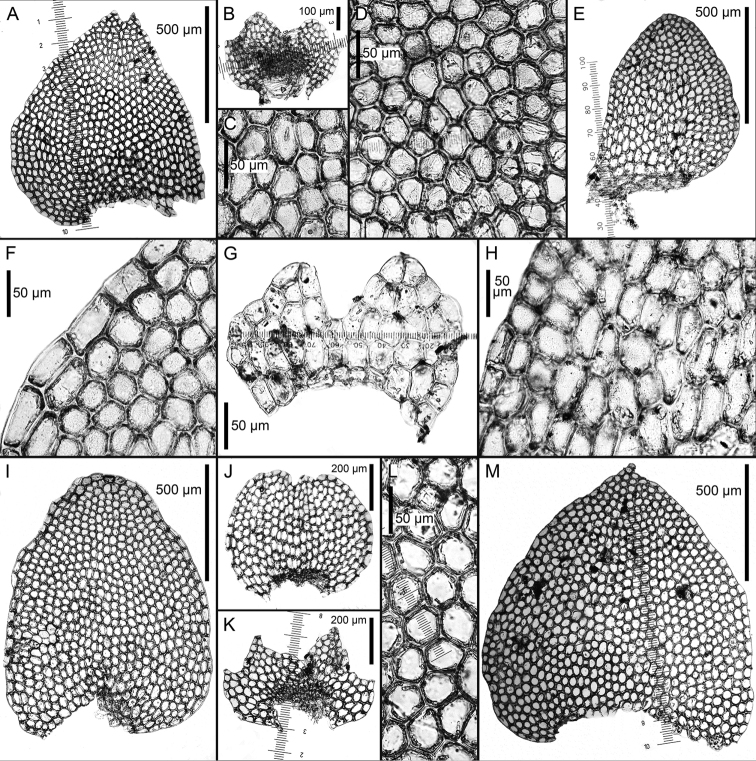
*Calypogeia latissima* Steph.: **A** leaf **B** underleaf **D** leaf middle cells *Calypogeia nasuensis* Inoue: **E** leaf **G** underleaf **H** leaf margin cells Calypogeia neesiana subsp. subalpina (Inoue) Inoue **F** leaf margin cells **I** leaf **J** underleaf *Calypogeia yoshinagana* Steph. **K** underleaf **L** leaf middle cells **M** leaf. Scale bars: 1 mm (**A, G, H**); 500 µm (**B, F, J, L, K**); 100 µm (**C, D, E)**; 50 µm (**I**). **A, B, D** from Lectotype G00061102; **E, G, H** from Holotype TNS-174632; **F, I, J** holotype NICH-49950; **K, L, M** from Lectotype G00067733.

Figures 10A–E , 11A, B, D

##### Type.

Philippines. Luzon, Merril (LECTOTYPE (designated here): G [G00061102!]).

##### Remarks.

The species was described from the Philippines (“Luzon”) and is very similar to the Meta-Himalayan *C. lunata*. Moreover, the translucent nature of plants and pale coloration may suggest the presence of blue oil bodies in living cells. Whether the difference in distribution is associated with the gap in genetics is not known. Currently, only the geographic concept may demonstrate the species status of the taxon.

There are two original specimens of the species in G. Both represent the parts of one original specimen (one was probably scheduled to be preserved in the Stephani herbarium, and the other should be returned to the collector) of which we prefer to select G00061102 as the lectotype because the second one (G00061101) has no original label (and is probable a duplicate).

The description from the lectotype is as follows: plants pale brownish (perhaps were bluish green when fresh), glistening and translucent, 1.5–2.0 mm wide; stem 180–230 µm wide, branching not seen; rhizoids common to numerous in brownish, loose, obliquely spreading fascicles; leaves contiguous or overlapping to 1/2 of leaf width, obliquely inserted and oriented, slightly convex, with apex commonly turned to ventral side, not decurrent, 800–1000 × 800–1000 µm, widely triangular-ovate, shortly bifid at the apex; underleaves obliquely spreading, decurrent for 1/3–1/2 of stem width, 1.2–2.2 as wide as stem, mostly bisbifid or with lateral tooth on one or on both sides, rarely bifid; cells in the midleaf 30–40 × 25–35 µm; nearly thin-walled, trigones small, cuticle nearly smooth in the leaf middle to very finely verruculose near leaf apices.

#### Calypogeia marginella

Taxon classificationPlantaeJungermannialesCalypogeiaceae

Mitt., J. Proc. Linn. Soc., Bot. 5 (18): 106. 1860 [1861].

BE8F4DC8-442F-5B18-9869-2D71B218B5D1

Figures 4T–Z, AA–AC, 8A–D

##### Type.

India. Khasia 1849 Hooker, no. 1339 (syntype: JE [JE-04005904, =JE-H4084!]; syntype: G [G00113555/5289!]).

##### Remarks.

*Calypogeia marginella* is a distinct narrow endemic taxon with a range probably restricted to the Khasia Hills. The species was described by Mitten (1860) from the Khasia Mountains (Hills). Singh and Nath (2007a) recorded it for the West Khasi Hills and East Khasi Hills. However, it is worth noting that the treatment of the taxon in Singh and Nath (2007a) should be incorrect because authors do not show in the figures nor mention in the description exceedingly large cells along leaf margin that are distinctly characteristic of the taxon. Which species they discussed under *C. marginella* is not clear to us.

The description based on the syntype G00113555/5289 is as follows: plants brownish to greenish brownish, slightly translucent and glistening, 2.0–2.5 mm wide; stem 140–200 µm wide, sparsely ventrally branched; rhizoids virtually absent or solitary, obliquely spreading; leaves contiguous to overlapping 1/3–1/2 of the leaf base of the next leaf, nearly planar to slightly convex, subhorizontally inserted and oriented, shortly or up to 1/2 of stem width decurrent, widely obliquely ovate to rounded-lingulate, with rounded apex, 1100–1300 × 1000–1300 µm; underleaves appressed to the stem, decurrent for 1/2–2/3 of stem width, divided mostly by very narrow V-shaped sinus into two lobes without additional lateral teeth or shortly bisbifid, with rounded to obtuse lobes, undivided portion 3–6 cell high; midleaf cells 30–65 × 17–37 µm, thin-walled, trigones small, cuticle smooth; marginal cells considerable larger and elongate along leaf margin, 70–80 µm long, with thickened external wall.

*Calypogeia marginella* is a very distinct species due to the elongated cells along the leaf margin, wide leaves and transversely elliptic but not deeply divided underleaves.

***Calypogeia nasuensis* Inoue, Bull. Natl. Sci. Mus. Tokyo, n.s. 12: 653. 1969.**

Figure 11E, G, H

##### Type.

Japan. Tochigi Prefecture: Nasu, 700 m a.s.l., August 1968, Empress Nagako (holotype: TNS: TNS-174632!] ; isotype: G [G00064238!]) .

##### Remarks.

The taxon is currently known from Japan only (Honshu). It was recently synonymized with *C. asakawana* (Isono et al. 2006), which is similar in relatively small underleaves and leaves with commonly rounded apices. However, we think these taxa are different due to the finely asperulose leaf cuticle, sometimes bisbifid underleaves and leaf apex not only rounded but also truncate and even shortly bifid, which are characteristic of *C. nasuensis* and dissimilar to the smooth cuticle, bifid underleaves, rounded leaf apex and less than 1/3 of stem width decurrent underleaves of *C. asakawana*.

The description based on the holotype is as follows: plants merely soft, glistening and translucent, greenish, 1.2–1.6 mm wide; stem 150–200 µm wide, sparsely ventrally branched; rhizoids numerous, in short, divaricate, grayish, erect spreading fascicles; leaves obliquely inserted and oriented, slightly concave-canaliculate or slightly convex (then apex somewhat turned to ventral side), ventrally decurrent for 1/2 of stem width or farther, 750–900 × 750–800 µm, with rounded or truncate apex; underleaves obliquely spreading, 1.0–1.5 as wide as stem, decurrent for 1/3–1/2 of stem width, deeply divided by U-shaped sinus into two lobes, entire at margin or with blunt tooth on one or each lateral side or very shortly bisbifid cells in the midleaf thin-walled, trigones vestigial to nearly absent, 35–52 × 25–40 µm, cuticle finely but distinctly papillose.

#### Calypogeia neesiana subsp. subalpina

Taxon classificationPlantaeJungermannialesCalypogeiaceae

(Inoue) Inoue, Mem. Natl. Sci. Mus. (Tokyo) 4: 58. 1971.

B6C1593D-7BBD-57AC-BDCA-6DD310E00082

Figure 11F–J

##### Basionym.

Calypogeia subalpina Inoue, J. Jap. Bot. 37 (4): 103. 1962.

##### Type.

Japan, Toyama Prefecture, Tateyama Mt., between Shishindake and Ryodake, 2600–2800 m a.s.l., on humus beneath *Pinus pumila* shrub, 15 August 1959, H. Inoue, no. 8733 (holotype: NICH [NICH-49950!]).

##### Remarks.

Unlike Calypogeia neesiana s. str., its subsp. subalpina may be expected in the eastern Sino-Himalaya. It differs from *C. neesiana* s. str. in larger marginal leaf cells (not only longer, as is typical for *C. neesiana*, but also wider, which is somewhat like marginal cells in *C. marginella*) and orbicular underleaves (versus underleaves transversely ellipsoidal).

The description based on the holotype is as follows: plants prostrate to loosely ascending, pale brownish in herbarium, forming loose mats, 1.1–2.0 mm wide and 5–10 mm long; rhizoids sparse to virtually absent, in several bundles obliquely to erect spreading or spreading up by the underleaf surface, from each underleaf base (if rhizoids developed), brownish to nearly colorless; stem brownish, 140–200 µm in diameter; leaves obliquely to subhorizontally inserted, dorsally insertion line transverse to arcuate, ventrally shortly decurrent, contiguous to subimbricate, ovate to obliquely ovate, 925–1075 × 625–875 µm; underleaves appressed to the stem, hyaline, 450–550 × 550–650 µm, nearly orbicular; midleaf cells subisodiametric to slightly oblong, ~25–40 µm in diameter, cells 5–6-gonal, thin-walled, with small and concave but distinct trigones, cuticle loosely verruculose; along margin 37–75 µm, with walls slightly thickened, trigones moderate in size, sometimes confluent on tangential side, concave; cells in underleaf middle mostly thin-walled with small to vestigial, concave trigones, along margin thin-walled, with small concave trigones.

#### Calypogeia udarii

Taxon classificationPlantaeJungermannialesCalypogeiaceae

Sudipa Das et D.K. Singh, Nelumbo 53: 194. 2011.

656790E7-6C29-5A1E-96CB-186989585A08

##### Type.

India. Eastern Himalaya: Arunachal Pradesh, Lower Dibang Valley district, Mehao Wildlife Sanctuary, Mayodia top, ~2850 m, 18 Nov 2000, D.K. Singh 98225 (holotype: BSD); not seen.

##### Remarks.

The species is known only from the type locality cited by Das and Singh (2011). The species is morphologically similar to *C. vietnamica*, although different in underleaves and leaf apices (oil body characteristics are not known in *C. udarii*), as discussed previously (Bakalin et al. 2019b).

#### Calypogeia yoshinagana

Taxon classificationPlantaeJungermannialesCalypogeiaceae

Steph. Bull. Herb. Boissier, sér. 2 8(9): 670. 1908.

CA836A95-ACD2-554C-A16F-9D78ABF85D21

Figures 10F–L , 11K–M

##### Type.

Japan. Mt. Yokogura, May 1901, T. Yoshinaga no. 38 (LECTOTYPE (designated here): G [G00067733!], another, poor specimen [G00282608!] is lectotype duplicate. This species was founded on the gatherings by T. Yoshinaga and U. Faurie, however only Yoshinaga’s collections are now present in G. Both reviewed specimens contain plants fully corresponding to the original description).

##### Remarks.

Hattori (1966) synonymized this species with *Calypogeia tosana*, regarding *C. yoshinagana* as only an environmentally induced modification. We, however, believe these are separate species. *Calypogeia yoshinagana* differs from *C. tosana* in acute leaves (very rarely bidentate and, if bidentate, the ‘lobes’ are distinctly unequal), more or less rigid texture, dull coloration (plants are not glistening). Attention to this species is needed in the eastern Meta-Himalayan flora where it may be revealed.

The description based on the lectotype is as follows: plants greenish brownish to dirty greenish, 1.8–2.2 mm wide, 2–3 cm long, relatively rigid; stem 200–250 µm wide, branching not seen; rhizoids sparse to common, in brownish, erect spreading fascicles; leaves obliquely inserted and oriented, slightly concave-canaliculate, leaves not decurrent ventrally, triangular-ovate, with acute or rarely obtuse or bidentate apices, 900–1100 × 1000–1200 µm; underleaves 1.5–2.5 as wide as stem, decurrent for 1/3–2/3 of stem width, clearly bisbifid, undivided area 2(–3) cells high; midleaf cells subisodiametric 30–50 × 27–40 µm, thin-walled, trigones small to very small, cuticle smooth.

### Key to *Calypogeia* taxa recorded for the Sino-Himalaya and eastern Meta-Himalaya or possibly expected there

**Table:** 

1	Leaf apex mostly rounded	**2**
–	Leaf apex acute to incised or distinctly bilobed	**12**
2	Underleaves shortly bilobed, emarginate or rounded at the apex	**3**
–	Underleaves distinctly bilobed, at least for 2/5 of the length	**5**
3	Underleaves as large as leaves or slightly smaller, leaves distinctly curved to ventral side, plants distinctly bluish when fresh due to blue (grading to purple!) oil bodies	***C. aeruginosa***
–	Underleaves much smaller than leaves, leaves not curved to ventral side, plants greenish to bluish greenish, oil bodies colorless to grayish	**4**
4	Cells along leaf margin elongate and distinctly wider than cells of intramarginal row	**C. neesiana ssp. subalpina**
–	Cells along leaf margin nearly isodiametric, smaller than in intramarginal row	***C. integristipula***
5	Cells along leaf margin distinctly swollen	***C. marginella***
–	Cells along leaf margin not different from intramarginal cell row	**6**
6	Underleaves bifid	**7**
–	Underleaves bisbifid or bifid with blunt tooth on each lateral side	**11**
7	Oil bodies biconcentric, with large central eye	***C. japonica***
–	Oil bodies never biconcentric	**8**
8	Leaves with mostly acute apex, only some leaves on some shoots narrowly rounded	**10**
–	Leaves with uniformly rounded apex	**9**
9	Underleaves decurrent for 1/3–1/2 of stem width, blunt teeth on lateral sides commonly present or underleaves bisbifid	***C. nasuensis***
–	Underleaves not or shortly decurrent (to 1/3 of stem width), without or rarely with additional blunt tooth on one side	***C. asakawana***
10	Stem ~1/5–1/6 of shoot width, underleaf lobes 8–10 cells in the base, underleaves 1.5–2.0 of stem width	***C. confertifolia***
–	Stem ~1/7–1/8 of shoot width, underleaf lobes 3–5 cells width in the base, underleaves 1.1–1.4 of stem width	***C. cuspidata***
11	Stem relatively narrow, ~1/8 of plant width, leaves nearly planar	***C. nasuensis***
–	Stem relatively wide, ~1/4 of plant with, leaves distinctly turned to dorsal side	***C. angusta***
12	Leaf apex acute	**13**
–	Leaf apex incised (sometimes shortly so) to distinctly bilobed	**25**
13	Leaf cuticle smooth, underleaves mostly distinctly wider than stem	**14**
–	Leaf cuticle very finely verruculose, underleaves as wide as stem or slightly wider	***C. apiculata***
14	Underleaves 1.1–1.5 as wide as the stem	**15**
–	Underleaves 1.5–3.5 as wide as the stem	**18**
15	Underleaf lobes 3–5 cells wide in the base, no additional lateral tooth on each side, leaves uniformly acute	***C. cuspidata***
–	Underleaf lobes more than 6–8 cells wide in the base, additional lateral teeth commonly present on one or both sides, leaves commonly shortly incised, rarely acute (at least some admixture of incised leaves present)	**16**
16	Underleaves commonly bisbifid, rarely with obtuse lateral teeth on both sides, oil bodies brownish blue to brownish, finely granulate	***C. granulata***
–	Underleaves commonly bifid with blunt (sometimes very smoothed) teeth on one or both sides, oil bodies not known	**17**
17	Underleaves decurrent for 1/3–1/2 of stem width, Hawaii	***C. cuspidata* [‘*C. hawaica*’ phase**]
–	Underleaves decurrent for 1/4–1/3 of stem width, Sri Lanka	***C. ceylanica***
18	Undivided portion of underleaf 2–3 cells high	**19**
–	Undivided portion of underleaf more than 4 cells high	**21**
19	Oil bodies colorless to grayish	**20**
–	Oil bodies deep blue to blue brown, coarsely granulate	***C. sinensis***
20	Underleaves commonly bisbifid	***C. yoshinagana***
–	Underleaves without lateral teeth	***C. confertifolia***
21	Underleaves bisbifid	**22**
–	Underleaves bifid	**23**
22	Underleaves decurrent for 0.5–1.0 of stem width, oil bodies blue, leaves sometimes shortly incised	***C. lunata***
–	Underleaves not or barely decurrent, oil bodies not known, leaves only acute	***C. khasiana***
23	Underleaf lobes obtuse, underleaves 1.8–2.5 times wider than stem	**24**
–	Underleaf lobes prominently acute, with 2–3 celled uniseriate ends, underleaves 3–4 times wider than stem	***C. vietnamica***
24	Underleaves divided by semicrescentic to U-shaped sinus, descending less than 1/7 of underleaf length	***C. formosana***
–	Underleaves divided by V- to U-shaped sinus descending for 1/3–2/5 of underleaf length (undivided portion of underleaves 3–5 cells high)	***C. cordistipula***
25	Midleaf cell surface finely verruculose	**26**
–	Midleaf cell surface smooth	**27**
26	Leaves constantly incised, cells in the leaf middle 40–80 × 30–60 µm, underleaves bisbifid	***C. arguta***
–	Leaves rarely incised, commonly apiculate, cells in the leaf middle 37–58 ×25–35 µm, underleaves bifid	***C. apiculata***
27	Underleaves bifid or with obscure additional teeth on one or both sides	**28**
–	Underleaves constantly bisbifid or with distinct and prominent additional lateral teeth on one or both sides	**31**
28	Underleaves 0.8–1.3 as wide as stem, its undivided portion 1–3 cells high	**29**
–	Underleaves 2.8–3.5 times as wide as stem, its undivided portion more than 5 cells high	***C. udarii***
29	Leaves apiculate to shortly incised into two strongly unequal or rarely nearly equal (sinus depth 2–3 cells) lobes	**30**
–	Leaves shortly bilobed, for two subequal lobes, leaf sinus depth 4–6 cells	***C. decurrens***
30	Leaf apex commonly obliquely truncate, unequally and very shortly bilobed, underleaves decurrent for 1/3 of stem width or less	***C. ceylanica***
–	Leaf apex mostly acute to obtuse, underleaves decurrent for 1/3–1/2 of stem width	***C. cuspidata* [‘*C. hawaica*’ phase**]
31	Undivided portion of underleaves 2–3 cells high, oil bodies blue to grayish, brown and colorless	**32**
–	Undivided portion of underleaves 4–5 and more cells high, oil bodies blue	***C. lunata***
32	Underleaves 1.1–1.3 as wide as stem, commonly bifid, with obscure additional lateral teeth on each side	***C. ceylanica*** [see also couplet 30]
–	Underleaves commonly more than 1.5 as wide as stem, almost constantly bisbifid	**33**
33	Leaves commonly acute, rarely incised (predominantly acute!)	**34**
–	Leaves commonly with incised apex, rarely acute	**35**
34	Oil bodies coarsely granulate, deep blue to blue-brown, plants merely soft, somewhat glistening, commonly wider 2.2 mm wide, leaves somewhat undulate at margins, commonly turned to ventral side	***C. sinensis***
–	Oil bodies not known, plants more or less rigid, not glistening, commonly less than 2.2 mm wide, leaves planar at margins, not turned to ventral side	***C. yoshinagana***
35	Oil bodies brownish to brown, blue and blue brown, botryoidal to granulate (in *C. latissima* not known but suspected as blue), leaves commonly incised at apex, sinus commonly V-shaped	**36**
–	Oil bodies colorless to grayish, botryoidal, leaves with almost constantly shortly divided apex by U-shaped sinus	***C. tosana***
36	Oil bodies blue to deep blue botryoidal or not known	**37**
–	Oil bodies brownish to brownish blue, finely granulate	***C. granulata***
37	Underleaves decurrent for 1/3–1/2 of stem width, oil bodies not known, leaves distinctly bilobed at apex (sinus depth 2–3 cells), leaves subimbricate	***C. latissima***
–	Underleaves not or barely decurrent, oil bodies presumably deep blue, leaves distinctly bilobed at apex (sinus depth 3–5 or more cells), leaves contiguous to distant	***C. goebelii***

## Phytogeographic speculations

The vertical movements of the Himalaya, Tibetan Plateau and Hengduan Mts. have influenced the speciation of various groups of biota, not only that of liverworts (Luo et al. 2014; Zhuo et al. 2013). These movements have additionally complicated the relationships within various groups and resulted in several phytogeographic boundaries crossing the eastern Sino-Himalaya. One of the most pronounced phytogeographic lines recognized today is the “Ward line” in the Salween-Mekong watershed (Luo et al. 2017). The robust differences between adjacent plant floras were formed due to uplift of the Qinghai-Tibetan Plateau and changes in river courses and correlated with increasing numbers and diversification of ecological niches (Clark et al. 2004; Shi et al. 1998). Niche diversification was associated with speciation. The same patterns were observed not only in plants but also in other groups of living organisms, e.g., birds (Cai et al. 2018). Moreover, the taxonomical diversity of taxa with narrow ranges in mountains could be explained by topography and evolutionary history, including geographic isolation rather than by the climate alone (Fjeldså and Rahbek 2006; Fjeldså et al. 2012; Jetz et al. 2004; Rahbek et al. 2007).

The eastern part of the Sino-Himalaya and the eastward adjacent Meta-Himalaya, as identified in this work, are valuable biodiversity hotspots on Earth (Myers et al. 2000; Luo et al. 2017). This general trend is also observed in *Calypogeia*, whose diversity is quite high in two respects: taxonomical and morphological. The data on the occurrence of *Calypogeia* taxa in the study area and nearby are placed in Fig. 1. The map indicates only reports where the geographic position of the collection might be identified with the deviations less than 400–500 km. In total, 11 taxa are known in the study area, and one more taxon (*Calypogeia marginella*) is found at a rather distant locality in the western Himalaya but may be expected in the study area. Two regularities in distribution are prominent: 1) all records in the study area and nearby are above 1000 m a.s.l., and 2) the annual amounts of precipitation in the collecting localities are between 1000 and 2000 mm per year. The exclusions are rare and belong mostly to *C. arguta* – a rather ‘weedy’ species of roadsides and other sites with disturbed vegetation cover. The third peculiarity has a presumptive character – this feature is the complete absence of taxa known and abundant in the boreal and hemiboreal Holarctic, including *C. integristipula*, *C. muelleriana*, *C. sphagnicola*, *C. orientalis*, etc. Although it is impossible to be absolutely sure that these taxa are absent from the Sino-Himalaya, the probability of occurrence of these species converges to zero.

Although 11 *Calypogeia* taxa are known within the study area, there are only three taxa restricted to this land: *C. cordistipula*, *C. sinensis* and *C. vietnamica*. However, for *C. aeruginosa* and *C. lunata*, the eastern Sino-Himalaya and eastern Meta-Himalaya are the area cores. *Calypogeia aeruginosa* is also known in southern Japan and Taiwan, where it is a possible relict. *Calypogeia lunata* spreads slightly southward of treated area, to northern Thailand. Other taxa are also distributed in the insular parts of East Asia, such as Japan and Taiwan (*C. angusta* and *C. granulata*), or slightly wider, in amphi-Pacific East Asia (*C. tosana* and *C. japonica*). Only *C. arguta*, as mentioned above, is a much more widely distributed taxon. The tight connection of amphi-Pacific floras with the Sino-Himalaya and Meta-Himalaya regions also implies that other taxa of *Calypogeia* presently known in insular and peninsular parts of East Asia and Southeast Asia and probably some other taxa known in South Asia may be expected in treated area.

In a broader context, taking into account the distribution of Calypogeiaceae in the Sino-Himalaya, the patterns can be found to be somewhat similar: Calypogeiaceae includes 5 genera (Söderström et al. 2016), of which the northern amphi-Pacific *Eocalypogeia* and Southeast Asian tropical *Mizutania* do not occur in the Sino-Himalaya and Meta-Himalaya. The merely speciose and antipodal *Mnioloma* has one species (and the only extratropical East Asian representative) distributed in northern Guizhou Province, China (Bakalin et al. 2015; Liu et al. 2013). *Metacalypogeia* has two species: the hemiboreal to cool-temperate Pacific-East Asian *Metacalypogeia cordifolia* (Steph.) Inoue and the mostly Sino-Himalayan *Metacalypogeia alternifolia* (Nees) Grolle that also reaches insular parts of East Asia.

## Conclusion

*Calypogeia* in the eastern Sino-Himalaya and Meta-Himalaya is still poorly understood taxonomically. The first attempt to summarize the information reveals that there are only a few data points based on a limited number of specimens. Moreover, many recorded taxa are poorly known, have questionable status or are presumably based on mistaken identifications. The taxa widely distributed in the North Holarctic (boreal zone and northward) are hardly possible in the study area, while the occurrence of some taxa from the south temperate zone of mountainous areas in southern Japan, Taiwan and the southeastern China mainland is quite probable. It seems that all, or nearly all, *Calypogeia* taxa of the Sino-Himalaya deeply penetrate to the eastern Meta-Himalaya and together form a highly peculiar pool of taxa reflecting the specificity of the Sino-Himalaya admitted in many biota groups. The identification key provided here is an attempt to increase research on and knowledge of *Calypogeia* in East Asia and should be further supplemented with exhaustive studies of living collections of the genus.

## Supplementary Material

XML Treatment for Calypogeia aeruginosa

XML Treatment for Calypogeia angusta

XML Treatment for Calypogeia apiculata

XML Treatment for Calypogeia arguta

XML Treatment for Calypogeia cordistipula

XML Treatment for Calypogeia granulata

XML Treatment for Calypogeia japonica

XML Treatment for Calypogeia lunata

XML Treatment for Calypogeia sinensis

XML Treatment for Calypogeia tosana

XML Treatment for Calypogeia vietnamica

XML Treatment for Calypogeia azurea

XML Treatment for Calypogeia fissa

XML Treatment for Calypogeia goebelii

XML Treatment for Calypogeia muelleriana

XML Treatment for Calypogeia neesiana

XML Treatment for Calypogeia sphagnicola

XML Treatment for Calypogeia trichomanis

XML Treatment for Calypogeia asakawana

XML Treatment for Calypogeia ceylanica

XML Treatment for Calypogeia cuspidata

XML Treatment for Calypogeia decurrens

XML Treatment for Calypogeia formosana

XML Treatment for Calypogeia integristipula

XML Treatment for Calypogeia khasiana

XML Treatment for Calypogeia latissima

XML Treatment for Calypogeia marginella

XML Treatment for Calypogeia neesiana subsp. subalpina

XML Treatment for Calypogeia udarii

XML Treatment for Calypogeia yoshinagana

## Footnotes

– About 80% of type specimens in liverwort herbarium in G have two codes: the first one was provided in pre-computer time, whereas the second one was provided in the digitalization process. The papers before 2000-th cited the only old number, whereas now it is better to cite the new one (linked with the picture in the database). However, we guess, the best choice is to cite both, because only in this case we may identify the numbers widely used before with the recent ones. Therefore, in our citation we use the new number first (sometimes only that, if no old number is available) and then, after slash, the old one.
